# The role of age in the physiological adaptations and psychological responses in bikini-physique competitor contest preparation: a case series

**DOI:** 10.1186/s12970-021-00445-1

**Published:** 2021-06-09

**Authors:** Daniel E. Newmire, Heather E. Webb

**Affiliations:** Department of Kinesiology, Exercise Physiology and Biochemistry Lab, Texas A&M University-Corpus Christi, Corpus Christi, TX 78412 USA

**Keywords:** Metabolism, Body composition, Skeletal muscle, Luteinizing hormone, Leptin

## Abstract

The increased popularity of the bikini-physique competitions has not translated to greater research identifying the influence of age on adaptations during contest preparation. The purpose of this case series was to observe how age may influence the adaptations normally seen during preparation and the exploration of newer protocols to address adaptations more relative to the judging standards. Over a 16-week pre-contest preparation, a 32-y bikini competitor (BC) and 44-y master’s bikini competitor (MBC) visited the laboratory bi-weekly to observe changes in body fat mass (BF), lean body mass (LBM), bone mineral density (BMD), total body water (TBW); exploratory measures of deltoid cross-sectional area (Delt_CSA_), gluteus maximus muscle thickness (GM_MT_), and subcutaneous adipose tissue thickness (SAT); reproductive hormones estradiol (E2), luteinizing hormone (LH), and energy balance hormones triiodothyronine (T_3_), leptin and ghrelin; hydration status during contest preparation and the week of competition; resting metabolic rate (RMR); psychometric data related to perceived anxiety, stress, and body image were assessed. No differences between BC and MBC were observed in BF, LBM, BMD, and TBW. Both competitors showed a small loss in LBM. Both BC and MBC showed a contrasting increase in Delt_CSA_ and a loss in GM_MT_. MBC showed to be slightly more dehydrated (1.025 vs 1.021 g·mL^− 1^) than BC. Both competitors maintained a euhydration status the day of the competition. No time differences were found between BC and MBC during RMR. BC showed a higher mean difference RMR compared to MBC (2.66 ± 0.75 kcal·kgLBM^− 1^·d^− 1^). MBC showed a higher mean difference in LH concentration (84.6 ± 6.01 IU·L^− 1^), which may be explained by perimenopausal status. MBC had a higher mean difference concentration of leptin (2.51 ± 0.24 ng·mL^− 1^·kgFM^− 1^), which was unperturbed by fat loss may be interrelated LH. BC self-reported a higher mean energy intake (15.07 ± 3.43 kcal·kgLBM^− 1^·d^− 1^) and higher aerobic training volume (93.26 ± 40.68 min·d). BC and MBC showed similar composition changes, slightly differing metabolic rates, and differing hormonal LH and leptin responses. This finding is in contrast to previous work showing both LH inhibition and leptin diurnal disturbance in younger, female athletes with low energy availability. The exploratory measures may have some benefit for bikini-physique competitors related to the judging criteria. Age did not seem to play a role in contest preparation adaptations.

## Introduction

In 2010, the International Federation of Bodybuilding (IFBB) formally recognized bikini competitions as an independent competition category in the competitive physique category. Since its introduction, the bikini category has grown to become to be a popular division on the fitness stage and due to its’ popularity, the bikini-physique category has expanded to include age-grouped competitions. Three age groups currently exist, including the master’s bikini-physique division (age ≥ 35 y), junior’s level (age 16–23 y), and the remaining category for contestants between 23 and 34 years of age. According to the National Physique Committee (NPC; amateur) and IFBB (professional), female competitors in the bikini division are judged on these criteria: 1) muscular shape, “*full roundness*” of gluteus maximus; 2) a lower body fat composition to distinctly present segregated gluteal and hamstring muscle groups; 3) a “*slight roundness*” of the deltoid muscle group; 4) a very lean, low body fat abdominal region (http://npcnewsonline.com/bikini-rules/).

It has been shown that exercise training has a positive impact on the health of middle-aged women. As women age into their perimenopausal stage, they experience a concurrent reduction in basal metabolic rate (BMR) and loss of lean body mass as they transition to menopause [1]. Changes in body composition (increased fat mass and decreased lean body mass) and in fat distribution (gynoid transition to android) seem to be influenced by the menopausal transition, as well as by chronological aging [1]. It has been shown that middle-aged women annually gain an average of 0.5 kg of fat mass or more [1]. This weight gain and reduction of BMR is accompanied by reduced physical activity, as women significantly reduce regular exercise during middle age by ~ 40% [2]. In contrast, active, middle-aged women tend to have an advantage as they enter the menopausal transition in terms of starting with a lower BMI, lower fat mass, greater lean mass, decreased risk of obesity, the higher associated increase in bone mineral density (BMD) in the femoral and spinal areas, and less android adiposity [1]. With the increased popularity of bikini-physique competitions and the known benefits of exercise training for middle-aged women, there is a need to investigate how contest preparation may influence adaptations in middle-aged females. To our knowledge, there are no current studies that have investigated and observed middle-aged female physique competitors to identify any notable impact age may have on contest preparation adaptations when compared to their younger cohort.

Recently, there has been a greater focus in observing how competition preparation impacts both physiological and psychological indices prior to and post-competition in bikini-physique competitors. However, this specific physique population has very limited data and co-analyzed with other competitor divisions in previous studies [3–11]. Hulmi, et al. observed 50 competitors (27.2 ± 4.1 y) over a ~ 20-week dieting phase followed by an 18-week recovery phase. Of the observed dieting phase group, 27 were IFBB amateur fitness competitors. Of these participants, 17 were bikini-physique competitors [12]. They found that the decreased energy intake in the diet group was mainly explained by the reduction of carbohydrate (CHO) intake with only very slight decreases in fat and no changes in protein intake. They observed that BF% decreased from 23.1 ± 5.6 to 12.7 ± 4.0% measured via DXA. Neither the diet nor recovery phase had much impact on changes in lean body mass (LBM) seen in the dieting group assessed by DXA. However, they did see a small decrease in ultrasound (US) assessed *vastus lateralis* CSA due to the diet phase. Hormone concentrations of leptin, testosterone, and triiodothyronine (T_3_) were reduced during the dieting phase [12].

Mathisen, et al. observed a categorically heterogeneous population of female physique competitors (28.1 ± 5.5 y). Of the 25 subjects competing, 21 were categorized as bikini-physique competitors (4 were defined as “body and fitness athletic” categories). The subjects were assessed three times during their contest preparation and compared to a similar non-contest preparation group of female physique competitors. The subjects were assessed at baseline, 2-weeks pre-competition, and 1-month post-competition. They assessed body composition via dual-energy x-ray absorptiometry (DXA) scan, resting metabolic rate (RMR) via indirect calorimetry, 4-day dietary recall and physical activity questionnaire, and psychometric data [11]. They found that competitors that did not use hormone contraceptives had greater menstrual irregularity than those that reported use. Dietary analysis showed that both groups’ CHO intake was below ACSM recommendations for moderate training athletes (5–7 g·kg^− 1^·d^− 1^) [13], yet within the ‘*realistic*’ range (2–5 g·kg^− 1^·d^− 1^) proposed by Roberts, et al. for physique athletes, which is dependent on the phase of training [14]. However, there is currently no established and widely accepted daily CHO intake recommendation for physique competitors. Resting metabolic rate (RMR) was suggested to be clinically low based on their comparative reference using Cunningham equation [15], which may suggest an energy deficiency. The contest preparation group showed the lowest RMR 2-weeks prior to their contest. Interestingly, the contest preparation group showed a slightly higher value of LBM compared to baseline at 2-weeks before the competition time point with a significantly lower reported kilocalorie (kcal) intake (33 g·kgLBM^− 1^·d^− 1^). The authors summarized that due to the low kcal intake reported (33 g·kgLBM^− 1^·d^− 1^) at 2-weeks prior to their contest was categorized as low energy availability (LEA) due to this value being uncorrected controlling for exercise energy expenditure from exercise training as previously recommended equation *Energy Availability (EA) = Energy Intake (EI) – Exercise Energy Expenditure (EEE)* [16]. The recommended threshold for maintenance for female athletes to maintain normal eumenorrhea is 30 g·kgLBM^− 1^·d^-1^ [17], the authors suggested that this may explain some of the changes they observed.

Longstrom, et al. recently published a post-contest focused outcome case series observing a sample population of four female-physique competitors (age 29.3 ± 4.9 y), that included two figure- and two bikini-physique competitors. In their analysis, they separated individual subject findings. They collected data at three-time points: 1–2 weeks pre-competition, 4 weeks, and 8–10 weeks post-competition. They assessed body composition using skinfold technique, total body water using multifrequency bioelectrical impedance analysis (MF-BIA), RMR, hormonal responses, muscular endurance, nutritional analysis, and subjective psychometric data that assessed sleep habits, quality of life, and menstrual cycle. They compared the change from the time point of 1–2 weeks prior to competition to 8–10 weeks post-competition. Focusing on bikini-physique competitors and between the time points of 1–2 weeks prior to competition and 8–10 weeks post-competition, they found that the adipokine hormone leptin that is synthesized from white adipose tissue, increased relative to the increase in fat mass gain. They also showed that the increase in kg fat mass increased total kg bodyweight and therefore was directly related to RMR. Lastly, when highlighting other hormonal changes, both T_3_ and thyroxine (T_4_) slightly increased from pre-competition measures.

As difficult as it is to accurately assess, report, and control in these observational studies, each of these previous investigations mentioned previously highlighted specific questions related to female-physique competitors. However, due to the inherent analysis of a heterogenic population of different categories of female competitors, limited sample collection time periods, and a comparing pre-competition status with post-competition status, it is difficult to discern more specific contest preparation responses to reference and compare for bikini-physique competitors. Each female-physique category will have different judging criteria, which may dictate the selected dieting and training protocols, and therefore the physiological and psychological responses. Additionally, some of the skeletal muscle measures used to assess the female-physique population [12] in these previous investigations have been well validated yet may lack an ability to generalize towards bikini-physique competitors relative to their judging criteria. Lastly, to our knowledge, there are currently no observational studies investigating the impact of contest preparation on masters-female competitors. The investigations mentioned previously recruited a demographically focused age range of ~ 23–34 y. The purpose of this case series was to observe, follow, and analyze physiological and psychological measures in two different age categories in female bikini-physique competitors preparing for a professional qualifying, national competition. In comparison to previous studies, our focus was an observational time course study during the contest preparation phase to compare any notable physiological and psychological differences between ages that could justify further studies with larger sample sizes. Additionally, our goal was to collect novel and exploratory measures that could be useful for this population based on the judging criteria for these contests.

## Materials and methods

### Participants and ethical approval

Two female bikini-physique participants (bikini competitor [BC]; master’s bikini competitor [MBC]) were recruited during this study. Unfortunately, due to the COVID-19 pandemic, numerous local, regional, and national contests during the Summer and Fall 2020 NPC competitive season were canceled, postponed, and/or relocated, thus reducing our ability to recruit and retain participants. The participants’ baseline characteristics are located below in Table 1. Both participants self-reported a regular menstruation cycle throughout the contest preparation. Additionally, both reported not using any form of birth control or pharmacological ergogenic aids. Supplement use was reported to be multivitamins, carnitine, and creatine for the BC and Vitamin E, Biotin, Zinc, Collagen, and Iron for MBC. This study was approved by the Texas A&M University-Corpus Christi Institutional Review Board (#*TAMU-CC-IRB-2020-02-027*), and the Institutional Biosafety Committee.

**Table 1:** Participant baseline characteristics

Participant Characteristics	BC	MBC
Age (y)	32	44
Height (cm)	163.2	156.2
Weight (kg)	55.9	53.07
Body Fat (%)	21.8	19.5
Body Fat Mass (kg)	11.71	9.98
Lean Body Mass (kg)	42.14	41.27
Skeletal Muscle Mass (kg)	21.22	19
Bone Mineral Density (g⋅cm^− 2^)	1.119	1.101
BMD Age Matched Z-Score (%)	106	105
RT Frequency (d⋅week^− 1^) ^a^	4–6	5–6
AT Frequency (d⋅week^− 1^) ^a^	6	6
Competition Experience (y) ^a^	3	2

Bikini-physique competitor (BC); Master’s bikini competitor (MPC); Bone mineral density (BMD); Resistance training (RT); Aerobic training (AT); ^a^denotes self-reported

### Experimental observational design

During both bi-weekly and monthly testing sessions, the participants visited the laboratory at 0800 every session after fasting for 8–10 h. Participants were instructed to not eat or drink before assessment and bring food and drink if needed to consume after testing procedures ended for that session. Testing procedures that occurred bi-weekly and monthly during the participant’s 16-week pre-contest preparation are shown in Table 2 in greater detail. Following the competition (week 16 to 20), the BC elected to end contest preparation to begin a reverse dieting protocol and lower the volume of exercise training. The MBC elected to continue contest preparation to compete in a subsequent competition (competition to week 20). Monthly procedures consisted of body composition via Dual X-ray Absorptiometry (iDXA) for body fat mass (FM), body fat % (BF%), lean body mass (LBM), and bone mineral content and density (BMC; BMD); ultrasound (US) for subcutaneous adipose tissue (SAT) thickness, and exploratory measures of deltoid cross-sectional area (Delt_CSA_) and gluteus maximus muscle thickness (GM_MT_), and total body water (TBW) was assessed with multifrequency bioelectrical impedance analysis (MF-BIA). Urine samples were collected upon their arrival for hydration status using urine specific gravity (USG) analysis. Venous blood sampling for hormone analysis took place post-RMR to minimize any impact of stress on metabolic rate measures. Finally, psychometric information using the Perceived Stress Scale; (PSS) [18], Body Image States Scale (BISS) [19], and dietary and exercise training recall data were also collected. Bi-weekly procedures included MF-BIA, hydration status via USG, psychometrics, and dietary and exercise training recall. Further, baseline and pre-competition values of body image and eating behaviors were collected using the Body Appreciation Scale (BAS-2) [20], Social Physique Anxiety Scale (SPAS) [21], and the Eating Attitudes Test 26 (EAT-26) [22]. The baseline values for both participants were 16-weeks from the competition they had originally planned.

**Table 2:** Data collection timeline

	Measure	Weeks										
		Baseline (1)	2	4	6	8	10	12	14	16	18	20
1	iDXA	X		X		X		X		X		X
2	MF-BIA	X	O	X	O	X	O	X	O	X	O	X
3	US	X		X		X		X		X		X
4	Blood Sample	X		X		X		X		X		X
5	Urine Sample	X	O	X	O	X	O	X	O	X	O	X
6	RMR	X		X		X		X		X		X
7	PSS & BISS	X	O	X	O	X	O	X	O	X	O	X
8	BAS-2, EAT-26, & SPAS	Z								Z		
9	Dietary Recall	X	O	X	O	X	O	X	O	X	O	X
10	Exercise Training Recall	X	O	X	O	X	O	X	O	X	O	X

Dual X-ray Absorptiometry (iDXA); Multi-frequency bioelectrical impedance analysis (MF-BIA); Ultrasound (US); Resting metabolic rate (RMR); Perceived Stress Scale (PSS); Body Image States Scale (BISS); Body Appreciation Scale (BAS-2); Eating Attitudes Test-26 (EAT-26); Social Physique Anxiety Scale (SPAS); X = Monthly data collection; O = Bi-weekly data; Z = Baseline and pre-contest collection; Dashed red line = competition

### Body composition analysis

The participant’s height and weight were collected prior to compositional measures using a digital scale and stadiometer (SECA 769; Chino, CA). Body composition was assessed with iDXA (iDXA, Lunar Prodigy; GE Healthcare, Madison, WI) following International Society for Clinical Densitometry protocol recommendations [23] to capture FM, BF%, LBM, and BMC. Furthermore, using a previous equation shown to be a reliable and accurate estimation of skeletal muscle mass (SKMM) by Kim, et al., we estimated SKMM from appendicular soft lean tissue (ASLT) [24]. To assess changes in body water, we utilized InBody 720 (InBody USA; Cerritos, CA). It has been previously shown that MF-BIA analysis has a R^2^ (0.82–0.86) and SEE (1.5–1.6 kg) in adult females when compared to the gold standard of total body water measure, isotopic deuterium dilution (D_2_O) [25]. Total body water was assessed bi-weekly and conveniently upon the return of the competitors 5-days post-competition to observe any notable fluid changes from post-competition hyperphagia similar to the state of weight recovery induced by post-starvation hyperphagia found in previous energy restriction studies [26].

Roughly, 80–90% of fat mass is stored subcutaneously [27]. to address local subcutaneous adipose tissue (SAT) changes directly, we utilized the US (GE Logiq E9 (GE Healthcare, Wauwatosa, WI) using a B-mode system with a wideband linear array transducer (L4-12t-RS) operating between 4.2–13 MHz with 12.7 × 47.1 mm footprint. Following ACSM protocol recommendations [28], the 7-site areas of the right side of the body were quantified following a previously published procedure [5]. The measurement sites included the chest, triceps, subscapular, midaxillary, suprailiac, abdomen, and anterior thigh, which were located using standard anatomical landmarks. The linear transducer was coated in water-soluble transmission gel which enabled acoustic contact without depression of the skin and SAT. The transducer was maintained perpendicular and two images were taken per site and SAT thickness was measured from the skin to the inside edge of the superficial fascia using NIH ImageJ software (http://imagej.nih.gov/ij/). Previous literature has validated (ICC = .99) using US for SAT analysis compared to MRI [29] and B-mode US has been validated to accurately assess SAT in a previous study [30]. Furthermore, the 7-site measures were used to estimate BF% using the appropriate equation to compare to iDXA [31]. Images were collected in duplicate at each of the 7 sites by the same US technician for every session. The inter-image SAT CV% was 4.28%.

### Exploratory regional skeletal muscle analysis

**Fig. 1:**
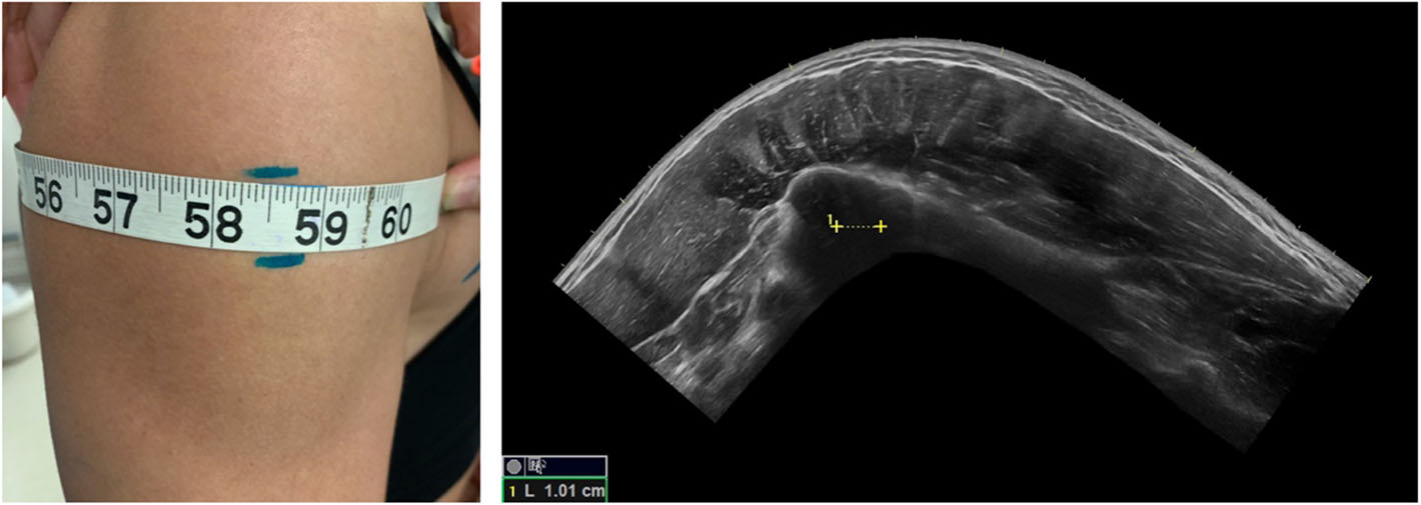
Cross-sectional area image of the deltoid (Delt_CSA_). The image on the left portrays the area of interest where the largest semi-circumference of the right deltoid was located and marked. Two lines were made above and below the tape measure. The transducer followed a path from the pectoralis major and the anterior deltoid interact to the furthest point posterior to capture the most medial portion of the posterior deltoid. The image on the right is the Delt_CSA_

The use of US to quantify muscular adaptations of both CSA and MT in the *vastus lateralis* has been validated to MRI [32–35]. However, the muscular adaptations in the regional area of the quadricep may be of less interest in the bikini-physique population we selected to observe. Based on the judging criteria listed above, both the muscular adaptations in the deltoid and gluteus maximus muscle groups may be of more importance and relative to meet bikini-physique judging requirements. Utilizing the same US unit, we acquired a panoramic LOGIQView® or extended field of view (EFOV) of the deltoid to assess cross-sectional area changes (Delt_CSA_). Due to the novelty of an exploratory nature of this measure, we collected 2 images of the largest semi-circumference of each competitor’s right arm, deltoid area (Fig. 1). The participant was instructed to sit on the examination table with arm relaxed at their side. Two lines were drawn to follow a path for the transducer to follow. The transducer was coated with a copious amount of water-soluble transmission gel and maintained perpendicular to the skin with minimal depression of the skin. Two images were taken at each session. Due to the complexity of the muscular anatomy segments of the deltoid and the quality of the US image, we were unable to discern definitive aponeuroses among anterior (A1, A2, A3) medial (M1), and posterior deltoid (P1, P2, P3) on all images. Due to this, we elected to quantify the deltoid CSA cumulatively. The Delt_CSA_ was analyzed using NIH ImageJ. The images were collected in duplicate by the same investigator. The inter-image of Delt_CSA_ CV% was .53%.

**Fig. 2:**
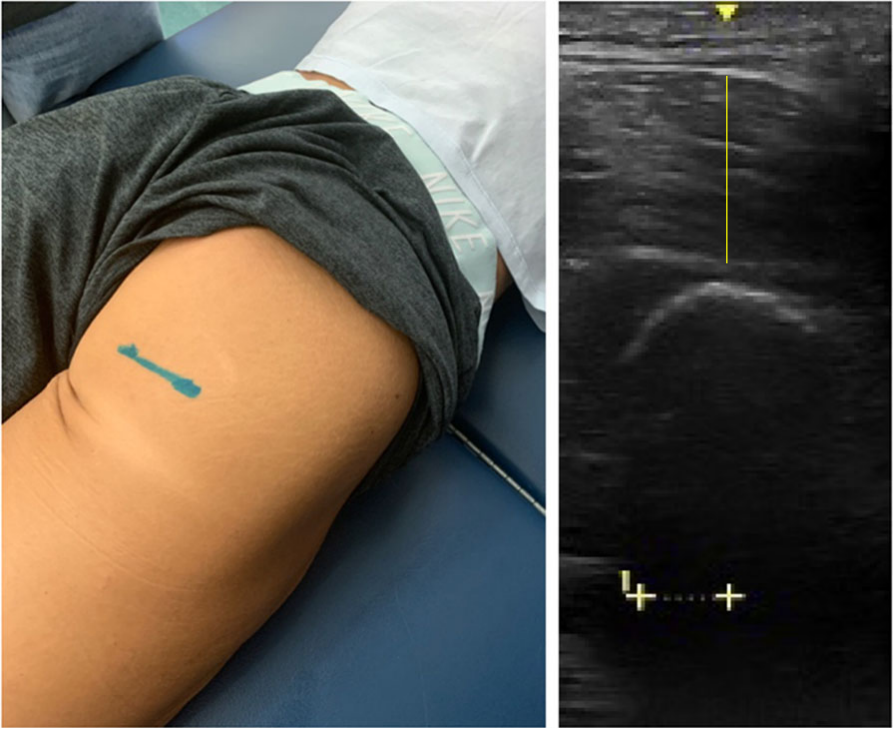
Gluteus maximus muscle thickness image (GM_MT_. The image of the left shows the marked location of the ischial tuberosity (IT). The image on the right shows the US measurement taken to assess GM_MT_

Additionally, we wanted to explore gluteus maximus (GM_MT_) adaptations during their contest preparation as this measure may hold a referring value to bikini-physique competitors and the contest judging requirements. Using a similar protocol in a previously published study exploring GM assessment with US [36], the participant was asked to lay prone and legs comfortably adducted. To determine the transducer placement, the examiners palpated from the participant’s posterior superior iliac spine (PSIS) to the ischial tuberosity (IT). After the IT was found, the participant was instructed to pull up their clothing, so their skin was available to again be palpated and the IT position was marked with a surgical pen (Fig. 2). Using B-mode imaging, the IT was found using a depth of 12 cm and two transverse images were taken in duplicate by the same investigator for every session. The GM_MT_ was measured in a straight line (90°) from the superficial aponeurosis of the GM_MT_ muscle, extending to the deep aponeurosis near the IT. The inter-image GM_MT_ CV% was .48%.

### Hydration status

Participants were asked to provide a urine sample after arrival to the laboratory or maintained in a cryogenic status during competition and transport back to the laboratory. Additionally, each participant traveled to their competition with urine collection cups to observe competition week and competition day hydration status. The participants were instructed to collect samples upon waking and place the sealed sample in a sealed plastic bag, and then store the sample in the freezer portion of a mid-sized refrigerator/freezer in their hotel suite. The 5-day competition week samples were then cryo-shipped in an insulated container back to the laboratory. Hydration status was assessed by urine specific gravity (USG) by aspirating 1–3 drops of the urine sample onto the lens of a digital scale clinical refractometer (Sper Scientific; Model 300,005; Scottsdale, AZ). The accuracy of the clinical refractometer is between ±0.002 and the refractive index changes proportionally to urine concentrations. Prior to urine USG analysis, both doubly distilled de-ionized water (DDH_2_O) and a prepared known density (1.020 g·mL^− 1^) of NaCl and DDH_2_O were analyzed in duplicate for reference values. Urine samples were analyzed in duplicate using standard procedures. Urinary USG sample values were then compared to the validated index of associated hydration status [37] values listed below in Table 3.

**Table 3:** Urinary specific gravity index for hydration status

Index for Hydration Status	
Condition	USG Value
Well-hydrated	< 1.010
Minimal dehydration	< 1.010–1.020
Significant dehydration	1.021–1.030
Serious dehydration	> 1.030

Urine Specific Gravity (USG); ≤1.020 is an indication of euhydration status [38]

### Resting metabolic rate

At each monthly session, resting metabolic rate (RMR) was assessed with indirect calorimetry (TrueOne 2400 Canopy System, ParvoMedics, Sandy, UT, USA). The TrueOne 2400 dilution mode system was calibrated before each assessment following manufacturer recommendations. Participants were asked to relax and lie supine on an examination table with the headrest set at an incline of ~ 45° during the 22-min assessment period and to maintain alertness with eyes open. The canopy was then placed over the head, shoulders, and upper chest of the participant to reduce environmental air to contaminating sample air entering or expired air escaping during measurement. The flow rate was established at ~ 28 to 30 ml·min^− 1^ within the first 1–3 min of the assessment as per the manufacturer’s instructions. The flow rate was adjusted to maintain the diluted CO_2_ percentage at ~ 1% during testing. The first 10 min of the testing procedure were discarded, and the following 12-min of the test were averaged. The percent between the measured RMR (RMR_Meas_) and the estimated RMR (RMR_Calc_) using the LBM Cunningham formula [15]. An RMR_Meas_/RMR_Calc_% between 90 and 110% is considered normal and is commonly used as a threshold for diagnosis of clinically low RMR, indicating energy deficiency [11, 39]. Measured RMR was expressed in both Kcal·d^− 1^ and Kcal·kgLBM^− 1^·d^− 1^ due to organ tissue being more metabolically active than skeletal muscle tissue in resting conditions, and LBM is considered a primary factor that explains a greater proportion of the variability in RMR [40].

### Dietary and exercise training recall

Participants followed guidance from a contest preparation coach. Self-reported bi-weekly dietary macronutrient intake data was collected using MyfitnessPal. Hand-held smartphone dietary tracking apps such as MyFitnessPal have shown to be more practical and a relative validity in comparison to other dietary recall procedures [41]. For exercise training recall, the investigators designed a training recall to collect data related to resistance training (RT) frequency and exercise selection. Additionally, participants were asked to report resistance exercise selected, sets, repetitions, and weight (lbs) to quantify RT volume (sets·reps) during contest preparation prior to their arrival to the laboratory [42]. For aerobic training (AT) recall, participants reported time, frequency, exercise selection, duration, and RPE of the bout, which was expressed in min·d. Due to the inherent difficulty of capturing the accurate self-reported intensity of AT bouts, we were unable to accurately estimate aerobic energy expenditure and how that may influence energy availability. Lastly, both BC and MBC were 93% compliant in self-reporting dietary and exercise training recall.

### Blood sampling and biochemistry analysis

After RMR assessment, a 4 mL blood sample was collected at each monthly session in K_2_ EDTA tubes that were acquired by a certified phlebotomist following WHO guidelines [43]. Samples were subsequently centrifuged at 3000 rpm at 4 °C for 10 min. Plasma samples (500 mL) were then transferred into storage microcentrifuge tubes and frozen at − 80 °C until later analysis. A small blood sample was used for hematocrit testing to determine any notable changes in plasma volume (PV%). Hormone analysis was completed utilizing readily available ELISA kits that included Estradiol (*MBS2606149*), Luteinizing Hormone (LH) (*MBS047228*), total T_3_ (*MBS580156*), leptin (*MBS020274*), and total ghrelin (*MBS3804142*). All hormones were expressed in the units supplied by the manufacturer. However, due to the hormone leptin being primarily synthesized by adipose tissue, leptin is also expressed per kg of FM. Intra-assay CV% concentration was found to be 6.82%. The mean PV% for each competitor was 47.12 ± 1.33%. It is to be noted that an early study hematocrit % analysis showed a competitor had a very low value and they were suggested to talk to their physician to make adjustments to increase their hematocrit to normal levels. No other issues were seen in hematocrit moving forward.

### Psychometrics

To explore the potential influence of self-perceived body image, stress, and eating behaviors on exercise behaviors, psychometric measurements were also collected, utilizing a computer-based survey system to collect this data. Baseline and pre-competition values of body image and eating behaviors were collected using the Body Appreciation Scale (BAS-2) [20], Social Physique Anxiety Scale (SPAS) [21], and the Eating Attitudes Test-26 (EAT-26) [22], while the Perceived Stress Scale (PSS) [18] and Body Image States Scale (BISS) [19] were collected during each bi-weekly data collection session. The BAS-2 was developed to assist in evaluating an individual’s perception of self-image. The BAS-2 is a ten-question, Likert-type scale on which statements are rated on a 5-point scale ranging from Never (receiving a score of 1) to Always (receiving a score of 5). The BAS-2 has valid psychometric properties, with previous studies reporting very good Cronbach’s α internal consistency values ranging from .87 to .93 in women [20].

Hart, et al. devised the SPAS to measures social anxiety related to an individual’s physique, specifically the body’s form and structure with a focus on body fat, muscular tone, and general body proportions, which mimic the desirable characteristics in the bikini-physique competition category. The SPAS is a 12-item self-report scale developed to assess the degree to which people become anxious when others observe or evaluate their physiques, on a scale of 1 (never) to 5 (always). The SPAS has high internal and test-retest reliability, with a Cronbach’s alpha coefficient of .90 and an eight-week test-retest reliability coefficient of .80 [21].

The BISS is a six-item measure of individuals’ evaluation and affects their physical appearance at a particular moment in time. Participants respond to six prompts on a 9-point, bipolar, Likert-type scales regarding satisfaction-dissatisfaction with their overall physical appearance, body size and shape, their weight, feelings of attractive or unattractiveness, current feelings of how they look compared to how they feel, and their appearance relative to the average person [19].

The PSS was asked bi-weekly to evaluate the extent of stress and lack of control that each participant had felt during that period. The PSS is a 10-item inventory scored on a Likert-type scale from 0 (never) to 4 (very often) about feelings and thoughts during the preceding month. Values on the PSS can range from 0 to 40, with increased values representative of greater perceptions of stress. Coefficient alpha reliability for the PSS was shown to be 0.84, 0.85, and 0.86 in three separate samples, with a test-retest correlation of 0.85 [18].

The EAT-26 [22] is a self-report questionnaire with 26-items that measure symptoms and concerns characteristic of eating disorders. The EAT-26 is scored using a six-point scale based on how often the individual engages in specific behaviors, ranging from always to never. Although the EAT-26 will yield a “referral index” based on three criteria, only the total score based on the answers to the EAT-26 questions was utilized in this study. Test-retest reliability for the EAT-26 ranges from 0.84 to 0.89 [44].

### Statistical analysis

Due to the inherent nature of this observational case series and the small sample number, the time course data was expressed in a descriptive nature to highlight any notable differences in the impact age may have on the adaptations seen during contest preparation in bikini-physique competitors. The observational and exploratory nature of this study and the outcomes highlighted may be used to assist in driving future studies with larger sample groups, rather than forming any definitive conclusions and relationships. Additionally, this data may assist both practitioners and bikini-physique competitors with a reference for contest preparation. Tables are presented changes from Baseline 1 to 16-weeks (pre-competition) were calculated in tables for unit change (△) and percent change (△%). Recommended or normal ranges were provided when appropriate for comparison from the ABIM Laboratory Test Reference Ranges and other qualifying references and organizations [13, 14, 17, 38, 39, 45–47]. Additionally, both the 4-day dietary recall and RMR data were expressed in both kcal·d^− 1^ and kcal·kgLBM^− 1^·d^− 1^ to control for participant LBM differences to compare to any notable recommendations. Traditionally, RMR is expressed kcal·d^− 1^ and compared between participants or to baseline. However, due to the variance of LBM on RMR measures [40], and skeletal muscle metabolism being a major determinant of RMR [48], data was additionally normalized to kg LBM to more fairly evaluate any meaningful differences. The macronutrients were expressed in g·kg^− 1^·d^− 1^ to compare to relative recommended intake ranges. Similar to LBM normalization, the hormone leptin was expressed per kg FM to control for any relative differences in fat mass between competitors [49]. The RT data was quantified and expressed in repetition volume (sets·reps). This repetition volume method was selected to reduce the embellishment of lower-body resistance training volume load due to greater load (kg) use, to more fairly compare volume between upper and lower body RT exercise volume and between competitors [42]. Data was expressed in mean ± SEM along with 95% confidence intervals (95% CI) where appropriate. Pearson’s r correlation coefficient was used when appropriate to highlight any potential relationships that assist in explaining outcomes and further generate future questions related to female-physique competitor studies. All statistical analysis and figure construction were done using GraphPad PRISM software (version 9.0; GraphPad Software Inc., San Diego, CA, USA).

## Results

### Body composition Figs. 3, 4, 5, 6 and 7, Table 4

**Fig. 3:**
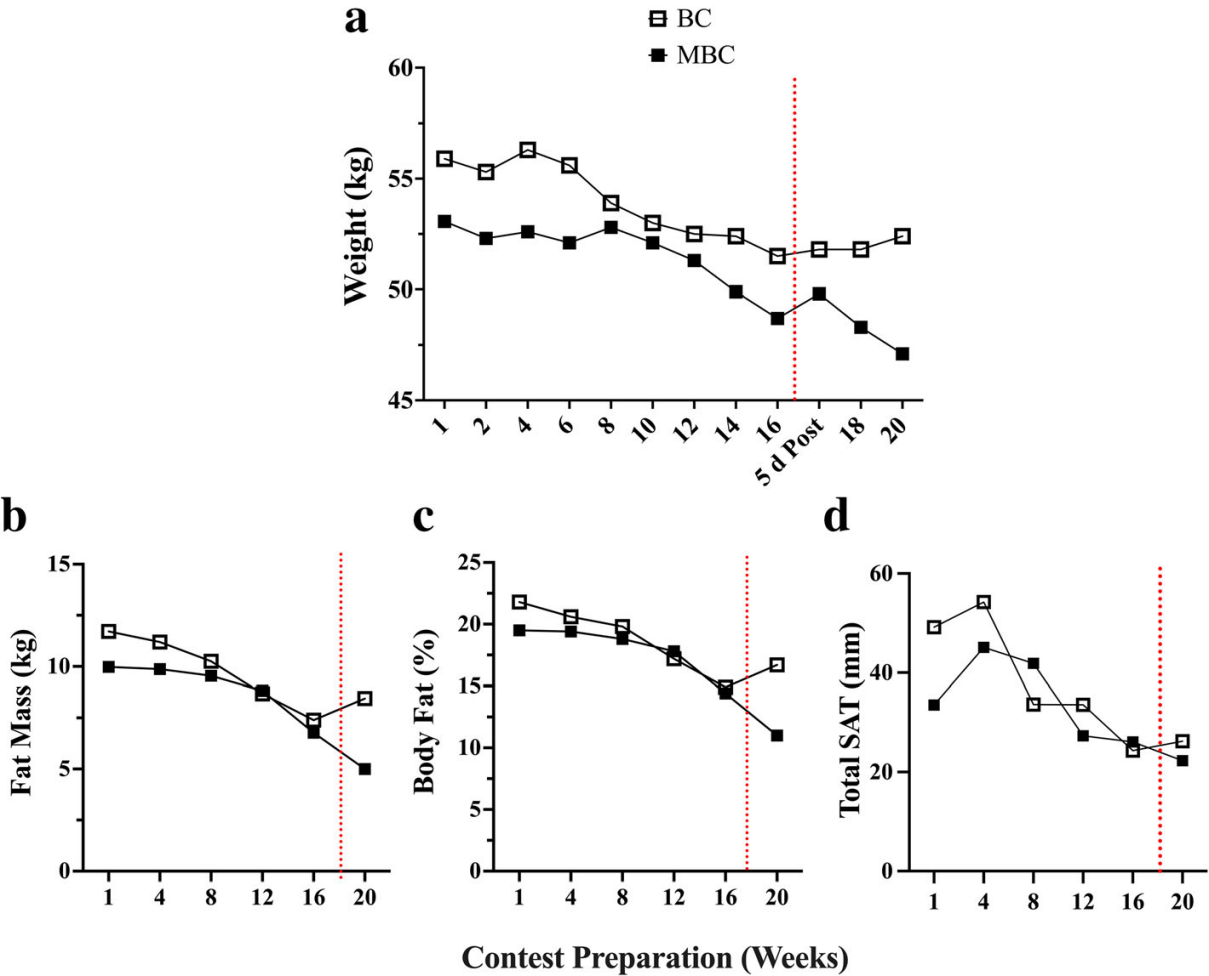
The time-course analysis of 20-week contest preparation with accompanying mean average (mean ± SEM; 95% CI) comparison between the BC and MBC on **a**) Weight, **b**) Fat mass, **c**) Body fat %, and **d**) Total subcutaneous adipose tissue (SAT). The BC had a higher mean body weight (53.5 ± 0.51; 95% CI: 52.4–54.66 kg) compared to the MBC (50.8 ± .58; 95% CI: 49.56–52.12 kg), higher mean fat mass (9.6 ± .69; 95% CI: 7.82–11.39 kg) compared to the MBC (8.33 ± .82; 95% CI: 6.21–10.45 kg), a higher body fat % (18.50 ± 1.07; 95% CI: 15.73–21.27%) compared to the MBC (16.82 ± 1.39; 95% CI: 13.23–20.40%), and a higher mean Total SAT (36.87 ± 4.98; 95% CI: 24.06–49.68 mm) compared to the MBC (32.69 ± 3.73 l 95% CI: 23.09–42.29 mm). The dashed red line denotes the competition

**Fig. 4:**
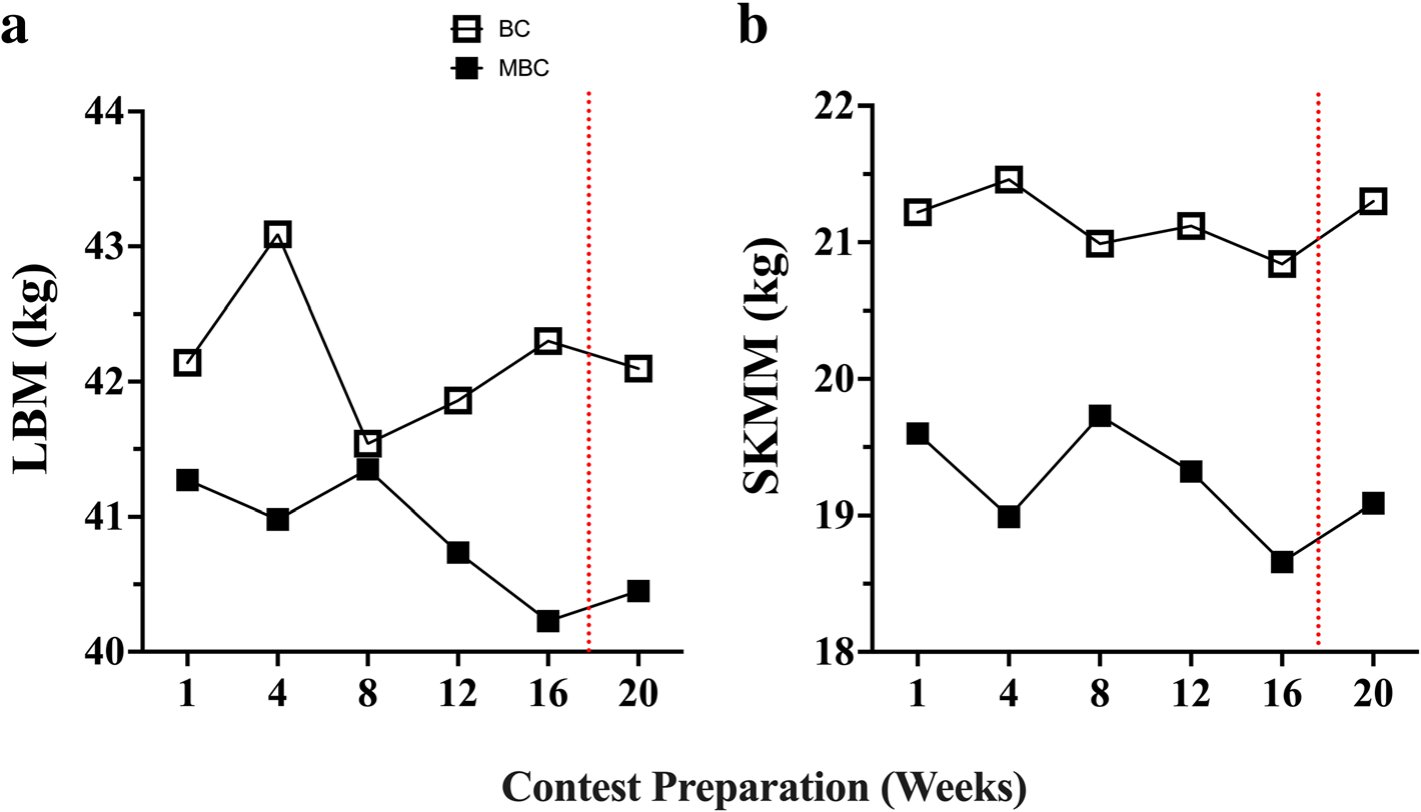
The time-course analysis of 20-week contest preparation with accompanying mean average (mean ± SEM; 95% CI) comparison between the BC and MBC on **a**) lean body mass (LBM), and **b**) estimated skeletal muscle mass (SKMM). The BC had a higher mean amount of LBM (42.17 ± .21; 95% CI: 41.62–42.72 kg) compared to the MBC (40.84 ± .18; 95% CI: 40.37–41.31 kg) and a higher estimated SKMM (21.16 ± .09; 95% CI: 20.92–21.39 kg) compared to the MBC (19.23 ± .16; 95% CI: 18.81–19.65 kg). The dashed red line denotes the competition

**Fig. 5:**
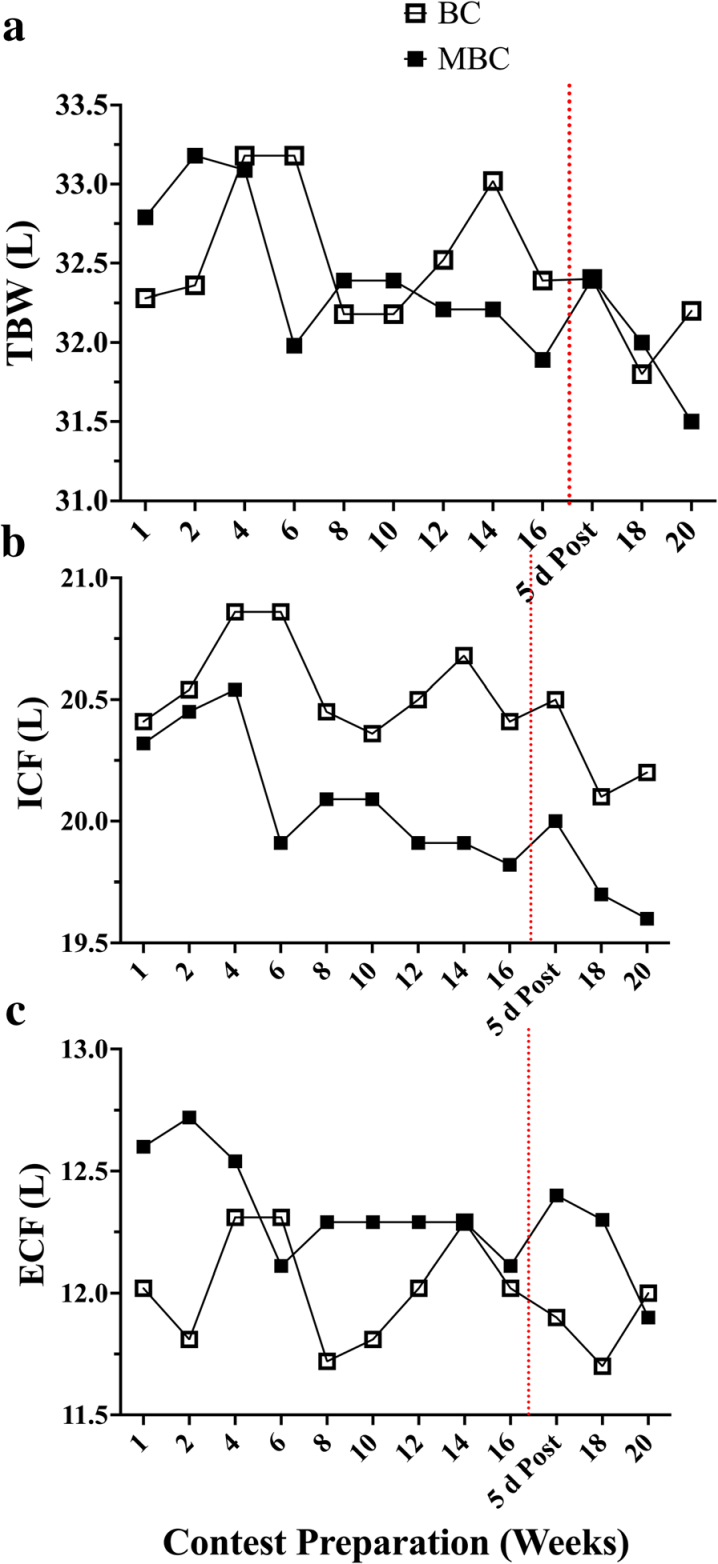
The time-course analysis of 20-week contest preparation with accompanying mean average (mean ± SEM; 95% CI) comparison between the BC and MBC on **a**) total body water (TBW), **b**) intracellular fluid (ICF), and **c**) extracellular fluid (ECF) with accompanying mean average (mean ± SEM; 95% CI) comparison between the BC and MBC. The BC has a slightly higher TBW (32.47 ± .12; 95% CI: 32.20–32.75 L) compared to the MBC (32.34 ± .14; 95% CI: 32.02–32.65 L) and ICF content (20.49 ± .06; 95% CI: 20.34–20.64 L) compared to the MBC (20.03 ± .08; 95% CI: 19.85–20.21 L). The MBC showed to have a higher ECF (12.32 ± .06; 95% CI: 12.18–12.46 L) compared to the BC (11.99 ± .06; 95% CI: 11.85–12.13 L). The dashed red line denotes the competition

**Fig. 6:**
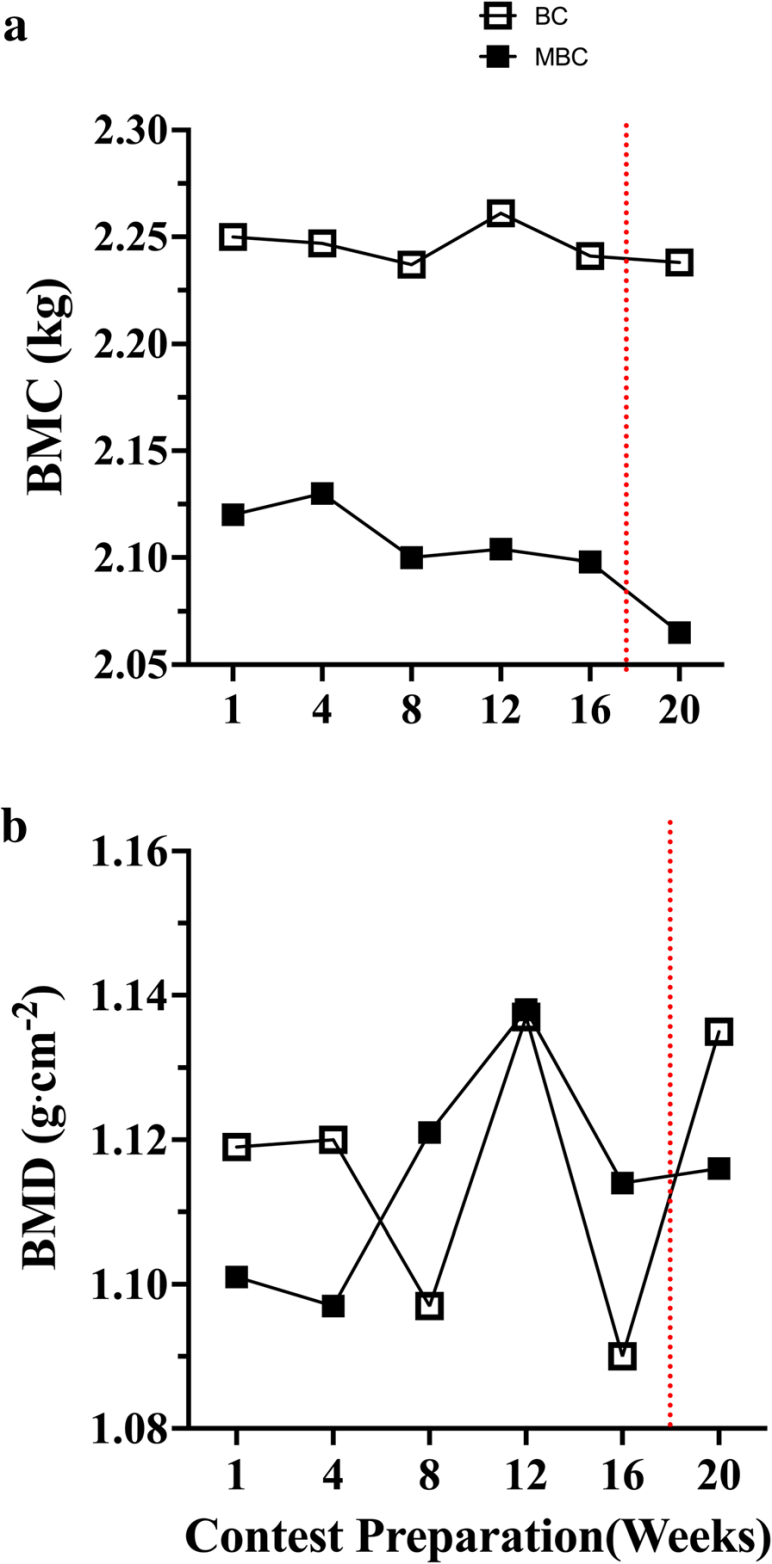
The time-course analysis of 20-week contest preparation with accompanying mean average (mean ± SEM; 95% CI) comparison between the BC and MBC on **a**) bone mineral content (BMC), and **b**) bone mineral density (BMD). The BC showed to have a slightly higher mean BMC (2.24 ± .003; 95% CI: 2.23–2.25 kg) compared to the MBC (2.10 ± .009; 95% CI: 2.07–2.12 kg). The BC and MBC showed to have similar mean BMD measures (1.11 ± .007; 95% CI: 1.09–1.13 g⋅cm^− 2^ vs 1.11 ± .006; 95% CI: 1.09–1.13 g⋅cm^− 2^). The dashed red line denotes the competition

**Fig. 7:**
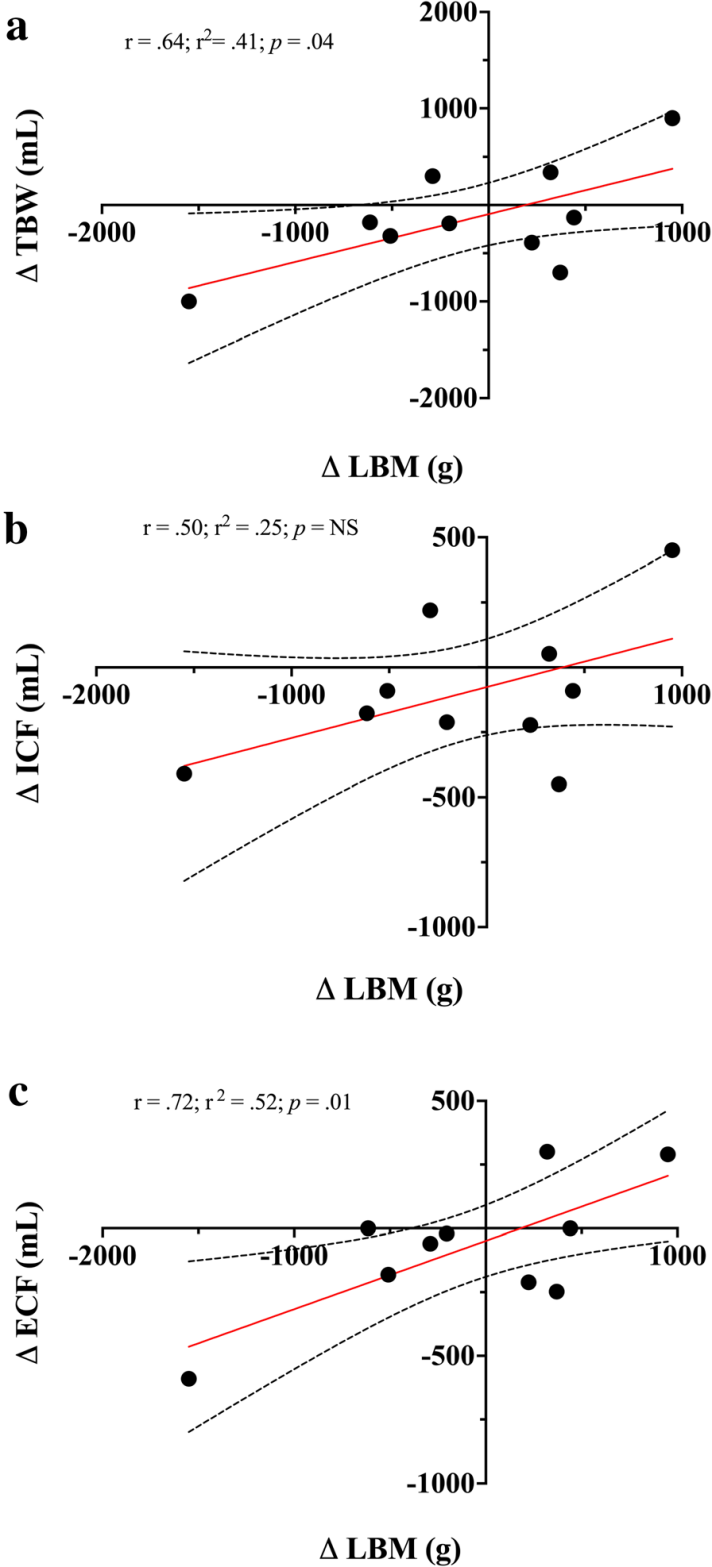
Assessing correlations between **a**) Δ total body water (TBW) and Δ lean body mass (LBM); **b**) Δ intracellular fluid (ICF) and Δ LBM; **c**) Δ extracellular fluid (ECF) and Δ LBM significant relationships were found between Δ TBW and Δ LBM (*p* = .04), and Δ ECF and Δ LBM (*p* = .01). NS was found for Δ ICF and Δ LBM

**Table 4:** Baseline to Week 16 Body Composition Changes

Body Composition	Recommended Range	Baseline	Pre-Contest (Week 16)	Δ Change	Δ% Change
**Bikini Competitor Contest Preparation**					
Weight (kg)	–	55.90	51.50	−4.40	−4.10
BMI (kg∙m^−2^)	18.5–24.9	20.99	19.34	−1.65	−4.10
Fat Mass (kg)	–	11.71	7.394	−4.32	− 22.59
Body Fat (%)	≥12	21.8	14.9	−6.90	−18.80
SAT Total (mm)	–	49.19	24.36	24.83	−33.76
LBM (kg)	–	42.14	42.301	0.16	0.19
SKMM (kg)	–	21.22	20.84	−0.38	−0.90
DeltCSA (cm^2^)	–	33.62	36.51	2.89	4.12
GMMT (cm)	–	4.21	3.284	−0.93	−12.36
BMC Total (kg)	–	2.25	2.241	− 0.01	−0.20
BMD Total (g∙cm^−2^)	–	1.119	1.090	−0.03	−1.31
TBW (L)	–	32.28	32.39	0.11	0.17
ECF (L)	–	11.81	12.02	0.21	0.88
ICF (L)	–	20.41	20.41	0.00	0.00
**Masters Bikini Competitor Contest Preparation**					
Weight (kg)	–	53.07	48.7	−4.37	−4.29
BMI (kg∙m-2)	18.5–24.9	21.75	19.96	−1.79	−4.29
Fat Mass (kg)	–	9.98	6.77	−3.21	−19.14
Body Fat (%)	≥12	19.5	14.4	−5.10	−15.04
SAT Total (mm)	–	33.54	26.07	−7.47	−12.52
LBM (kg)	–	41.27	40.23	−1.04	−1.28
SKMM (kg)	–	19.6	18.66	−0.94	−2.46
DeltCSA (cm2)	–	26.71	29.60	2.89	5.14
GMMT (cm)	–	3.97	2.29	−1.68	−26.77
BMC Total (kg)	–	2.12	2.098	−0.02	−0.52
BMD Total (g∙cm-2)	–	1.101	1.114	0.01	0.59
TBW (L)	–	32.79	31.89	−0.90	−1.39
ECF (L)	–	12.72	12.11	−0.61	−2.46
ICF (L)	–	20.32	19.82	−0.50	−1.25

Subcutaneous adipose tissue (SAT); Lean body mass (LBM); Skeletal muscle mass (SKMM);

Deltoid cross-sectional area (DeltCSA); Gluteus maximus muscle thickness (GMMT)

Bone mineral content (BMC); Bone mineral density (BMD); Total body water (TBW);

Extracellular fluid (ECF); Intracellular fluid (ICF)

### Hydration status Fig. 8, Table 5

**Fig. 8:**
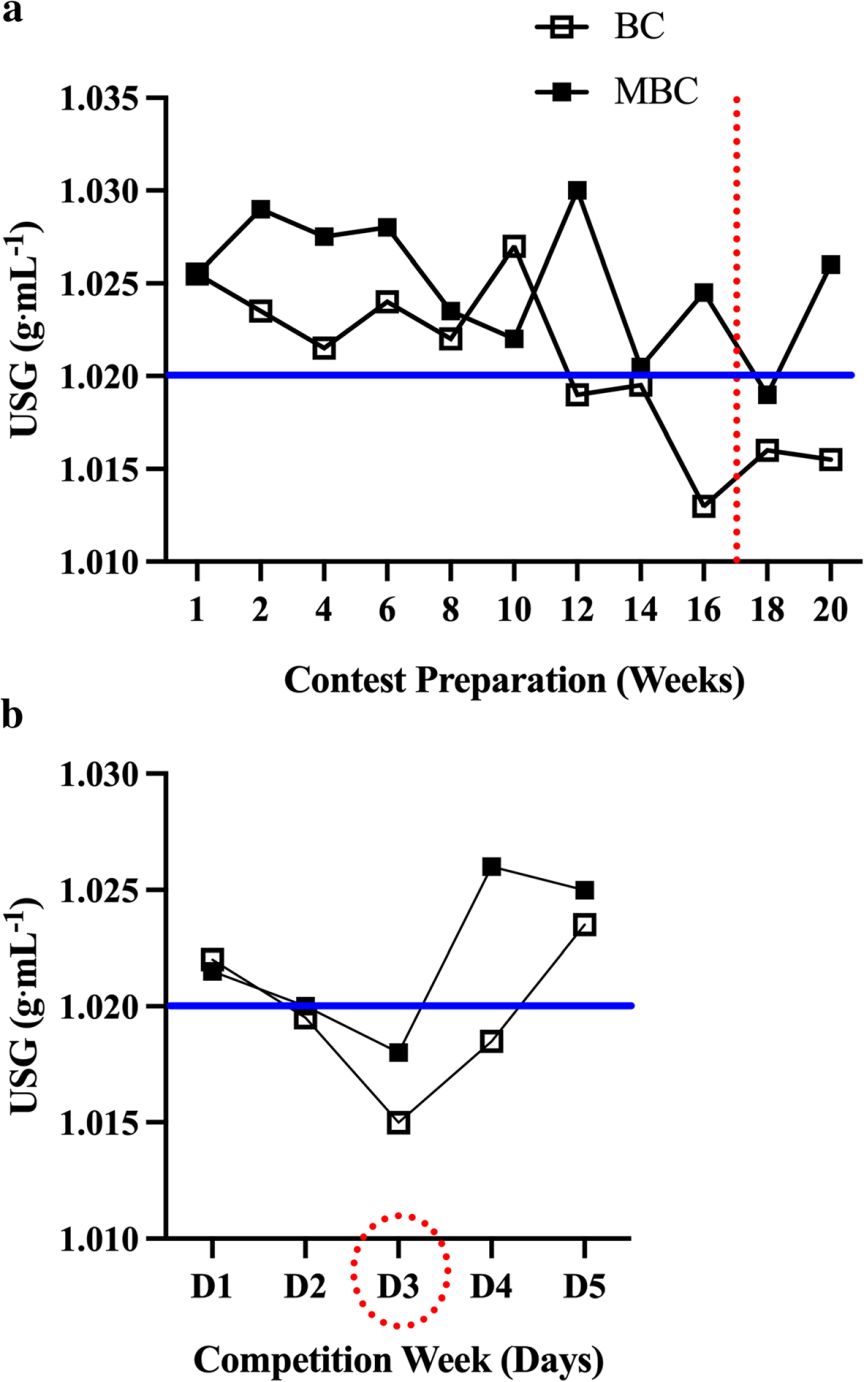
The time-course analysis of 20-week contest preparation of hydration status assessed with urine specific gravity (USG) with accompanying mean average (mean ± SEM; 95% CI) comparison between the BC and MBC during **a**) Contest Preparation (20 weeks) and (**b**) Competition week time course analysis assessed with over 5 d (D1-D5). The mean hydration status during contest preparation showed that the MBC had a higher value (1.025 ± .001; 95% CI: 1.023–1.027 g⋅mL^− 1^) compared to the BC (1.021 ± .001; 95% CI: 1.018–1.024 g⋅mL^− 1^). During the competition week, the MBC also had a higher value (1.022 ± .001; 95% CI: 1.018–1.026 g⋅mL^− 1^) compared to the BC (1.020 ± .001; 95% CI: 1.016–1.024 g⋅mL^− 1^). The dashed red line and circle denote the competition. The solid blue line denotes the euhydration threshold (1.020 g·mL^− 1^)

**Table 5:** Hydration Baseline to 16-week Change

Hydration Status	Recommended Range	Baseline	Pre-Contest (Week 16)	Δ Change	Δ% Change
**Bikini Competitor Contest Preparation**					
USG	≤1.020	1.0255	1.013	−0.01	− 0.61
**Masters Bikini Competitor Contest Preparation**					
USG	≤1.020	1.0255	1.0245	0.00	−0.05

Urine specific gravity (USG)

### Resting metabolic rate Fig. 9

**Fig. 9:**
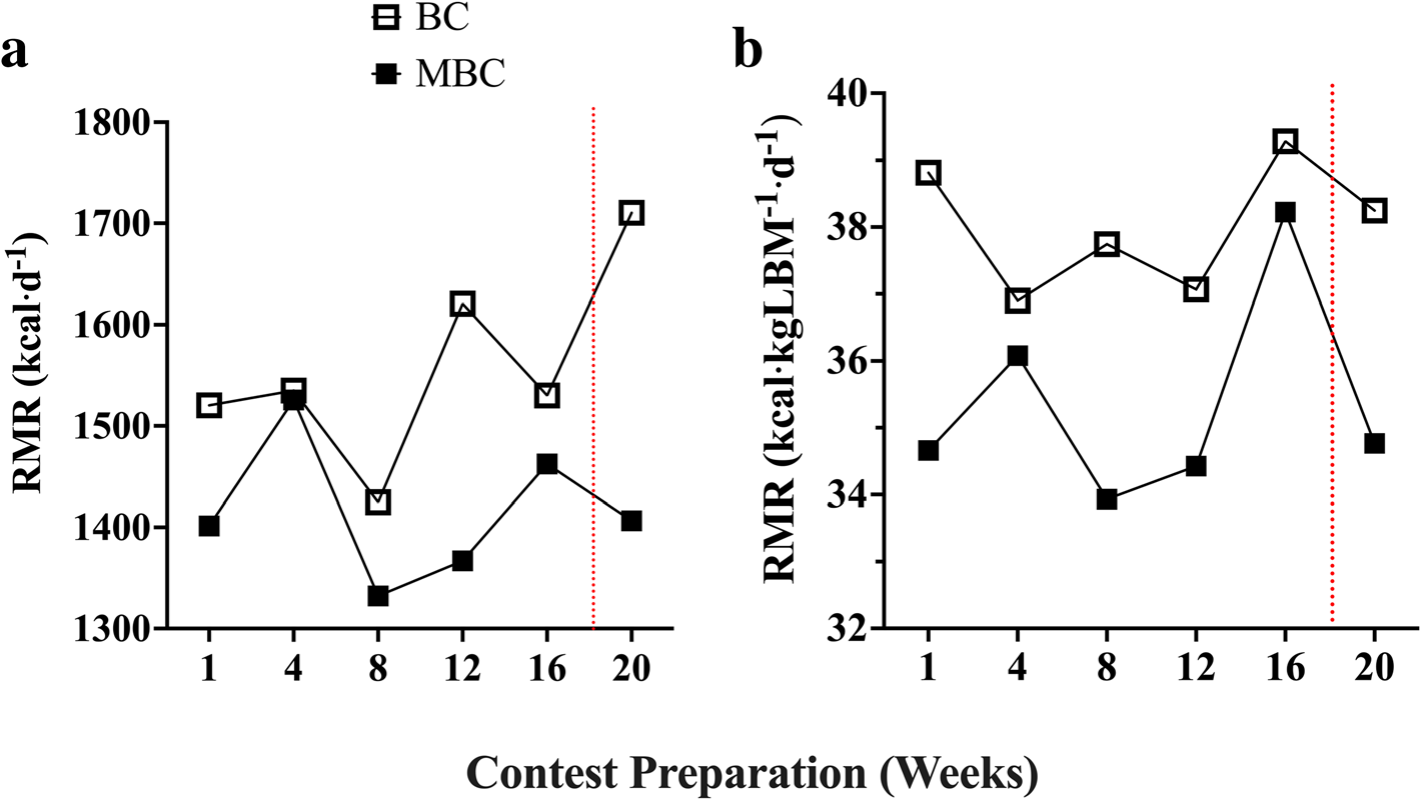
Exploratory assessment of the time course of RMR during 20-week contest preparation with accompanying mean average (mean ± SEM; 95% CI) comparison between the BC and MBC on **a)** RMR expressed in kcal·d^− 1^ and **b)** RMR expressed in kcal·kgLBM^− 1^·d^− 1^. The BC had a higher mean RMR rate in both analyses. The traditional expression (BC: 1557 ± 39.92; 95% CI: 1454–1660 kcal⋅d^− 1^ vs MBC: 1416 ± 28.25; 95% CI: 1343–1489 kcal⋅d^− 1^) and RMR normalized to kg LBM (BC: 39.66 ± .52; 95% CI: 38.32–41.00 kcal·kgLBM^− 1^·d^− 1^ vs MBC: 37.80 ± .34; 95% CI: 36.92–38.67 kcal·kgLBM^− 1^·d^− 1^). The red dashed line denotes the competition

### Energy intake and exercise training recall Figs. 10 and 11, Table 6

**Fig. 10:**
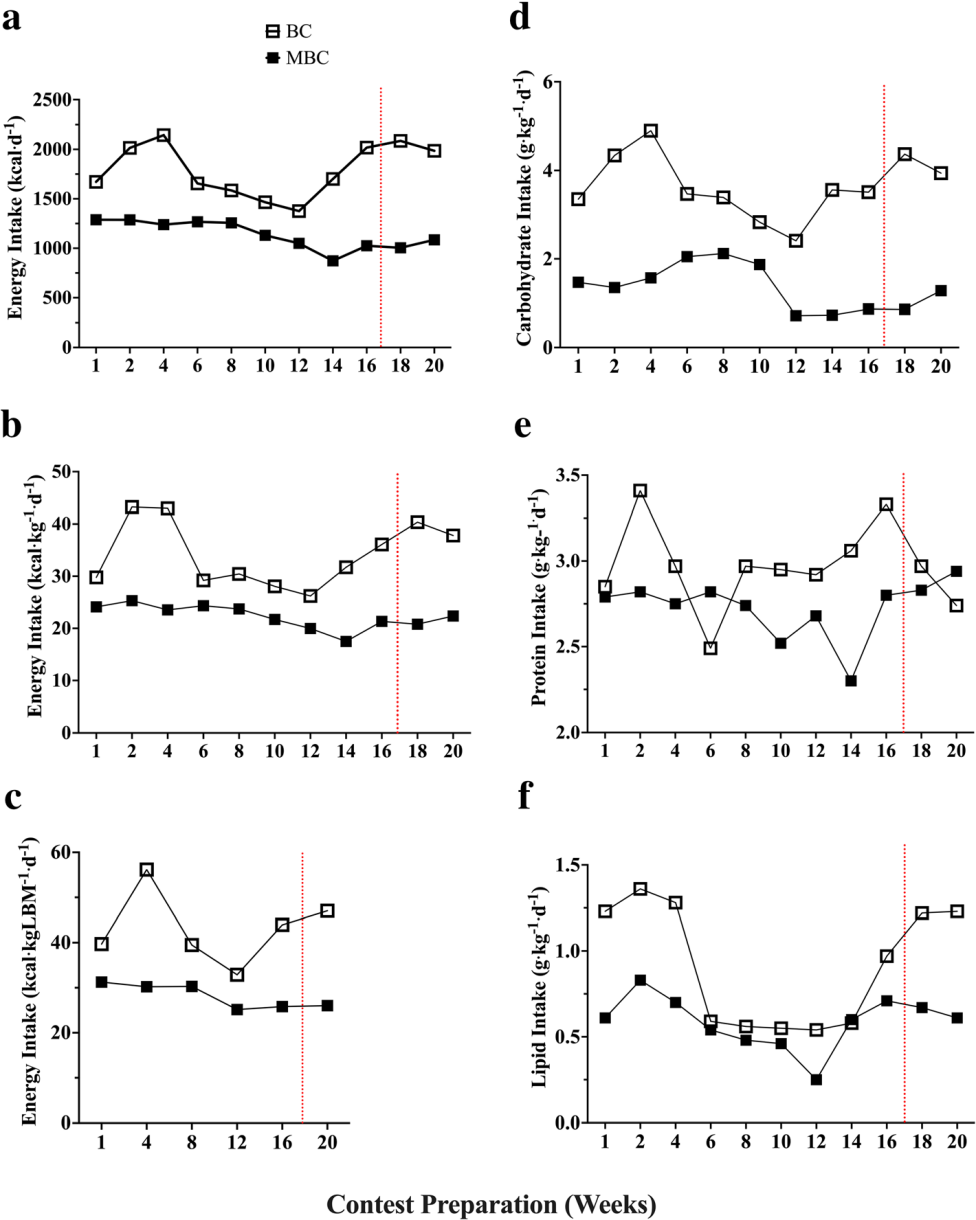
The time course analysis of 20-week contest preparation for energy intake from the self-reported 4-day dietary recall (kcal·kg^− 1^·d^− 1^ and) with accompanying mean average (mean ± SEM) comparison between BC and MBC. **a**) Energy Intake expressed in kcal·d^− 1^, **b**) Energy Intake expressed in kcal·kg^− 1^·d^− 1^, **c**) Energy Intake expressed in kcal·kgLBM^− 1^·d^− 1^, **d)** Carbohydrates (g·kg^− 1^·d^− 1^), **e)** Protein (g·kg^− 1^·d^− 1^), and **f)** Lipids (g·kg^− 1^·d^− 1^). The BC had a higher energy intake in all three expressed analyses (BC: 1791 ± 80.45; 95% CI: 1612–1971 vs MBC: 1137 ± 42.35; 95% CI: 1043–1232 kcal·d^− 1^; BC: 34.18 ± 6.16; 95% CI: 30.04–38.31 vs MBC: 22.25 ± 2.28; 95% CI: 20.71–23.78 kcal·kg^− 1^·d^− 1^; BC: 43.20 ± 3.24; 95% CI: 34.86–51.55 vs MBC: 28.13 ± 2.73; 95% CI: 25.26–31.01 kcal·kgLBM^− 1^·d^− 1^). The BC had a higher mean carbohydrate intake (BC: 3.64 ± .21; 95% CI: 3.16–4.12 vs MBC: 1.35 ± .15; 95% CI: 1.00–1.70 g·kg^− 1^·d^− 1^, higher mean protein intake (BC: 2.96 ± .07; 95% CI: 2.8–3.13 vs MBC: 2.72 ± .05; 95% CI: 2.60–2.84 g·kg^− 1^·d^− 1^), and a higher lipid intake (BC: .91 ± .1; 95% CI: .6–1.15 vs MBC: .58 ± .04; 95% CI: .48–.69 g·kg^− 1^·d^− 1^). The red dashed lines denote competition

**Fig. 11:**
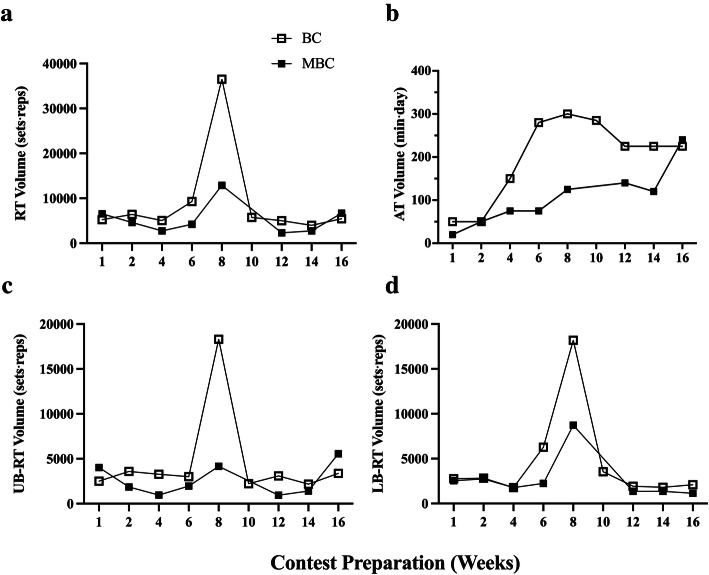
Time course analysis during 16-week pre-contest preparation prior to competition of self-reported resistance training (RT) volume (sets⋅reps⋅day), and aerobic training (AT) volume (min⋅d) with accompanying mean average (mean ± SEM) comparison between BC and MBC. The BC and MBC self-reported an average frequency of 4.8 ± .11 and 3.8 ± .22 d respectively of RT bouts prior to arriving to the lab for each session. **a**) The Total RT volume, **b**) The aerobic training (AT) volume, **c**) The upper body (UB) RT volume, **d**) The lower body (LB) RT volume. The BC had a higher mean total mean RT volume (9194 ± 3499; 95% CI: 1242–17,147 sets⋅reps) compared to MBC (5345 ± 1230; 95% CI: 2435–8254 sets⋅reps), a higher mean AT volume (198.9 ± 31.84; 95% CI: 125.5–272.3 min⋅d) than the MBC (105.6 ± 67.74; 95% CI: 48.99–162.3 min⋅d), a higher mean UB-RT volume 4619 ± 1720; 95% CI:652.5–8585 sets⋅reps) than the MBC (2609 ± 611.5; 95% CI:1163–4055 sets⋅reps), and a higher mean LB-RT volume 4576 ± 1765: 95% CI: 504.9–8646 sets⋅reps) than the MBC (2736 ± 881.3; 95% CI: − 651.8-4820 sets⋅reps). The red dashed lines denote competition

**Table 6:** Energy Status Baseline to 16-week Change

Energy Status	Recommended Range	Baseline	Pre-Contest (Week 16)	Δ Change	Δ% Change
**Bikini Competitor Contest Preparation**					
Energy Intake (kcal∙kgLBM-1∙d-1)	30–45 kcal∙kgLBM-1∙d-1	39.69	43.95	4.26	5.09
Carbohydrate Intake (g∙kg-1∙d-1)^a^	2–5 (g∙kg-1∙d-1)	3.35	3.51	0.16	2.33
Lipid Intake (% of Total kcal∙d-1)	> 20% Total kcal∙d-1	41	24.1	−0.17	−25.96
Protein Intake (g∙kg-1∙d-1)	1.2–2.0 g∙kg-1∙d-1	2.85	3.33	0.48	7.77
Measured RMR (kcal∙d-1)	–	1520	1530	10.00	0.33
Cunningham Estimated RMR (kcal∙d-1)	–	1427.08	1430.62	3.54	0.12
RMRMeas/RMRCalc %	90–110%	106.51	106.95	0.44	0.20
Measured RMR (kcal∙kgLBM-1∙d^1^)	–	38.82	39.29	0.47	0.60
**Masters Bikini Competitor Contest Preparation**					
Energy Intake (kcal∙kgLBM-1∙d-1)	30–45 kcal∙kgLBM-1∙d-1	31.26	26.04	−5.22	−9.11
Carbohydrate Intake (g∙kg-1∙d-1)^a^	2–5 (g∙kg-1∙d-1)	1.47	0.87	−0.60	−25.64
Lipid Intake (% of Total kcal∙d-1)	> 20% Total kcal∙d-1	30.9	29.9	−0.01	−1.64
Protein Intake (g∙kg-1∙d-1)	1.2–2.0 g∙kg-1∙d-1	2.79	2.8	0.01	0.18
Measured RMR (kcal∙d-1)	–	1401	1462	61.00	2.13
Cunningham Estimated RMR (kcal∙d-1)	–	1407	1385	−22.00	−0.79
RMRMeas/RMRCalc %	90–110%	99.57	105.56	0.06	2.92
Measured RMR (kcal∙kgLBM-1∙d-1)	–	34.66	38.23	3.57	4.90

Lean body mass (LBM); Resting metabolic rate (RMR); Measured (Meas); Calculated (Calc)

^a^Carbohydrate intake recommendations for physique athletes Roberts et al., [14]

### Hormone analysis Fig. 12

**Fig. 12:**
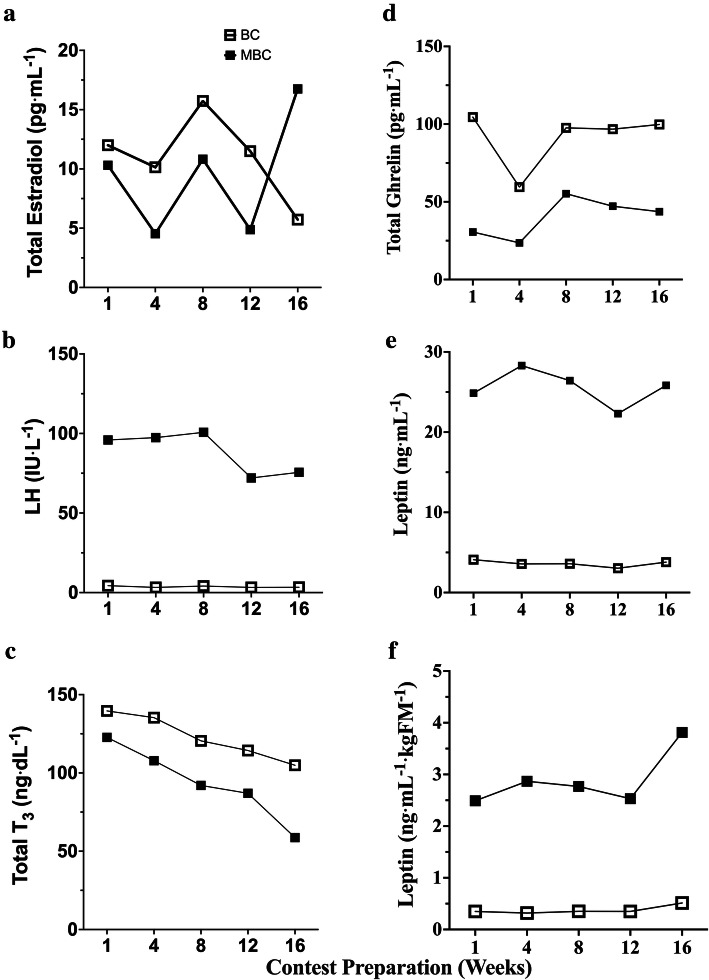
Time course analysis during 16-week pre-contest preparation of reproductive, metabolic, and energy balance hormones with accompanying mean average (mean ± SEM) comparison between BC and MBC on **a**) Total Estradiol (E2) (pg⋅mL^− 1^), **b**) Luteinizing hormone (LH) (IU⋅L^− 1^), **C**) Total Triiodothyronine (T_3_) (ng⋅dL^− 1^), **d)** Total Ghrelin (pg⋅mL^− 1^), **e)** Leptin (ng⋅mL^− 1^), and **f)** normalized leptin to kg body fat (ng⋅mL^− 1^⋅kgFM^− 1^). The BC showed to have a higher mean E2 concentration (11.02 ± 1.61; 95% CI: 6.53–15.5 pg⋅mL^− 1^) compared to MBC (9.45 ± 2.24; 95% CI: 3.22–15.69 pg⋅mL^− 1^). The MBC showed to have a higher mean LH concentration (88.34 ± 6.01; 95% CI: 71.65–105.0 IU⋅L^− 1^) compared to the BC (3.66 ± .23; 95% CI: 3.00–4.31 IU⋅L^− 1^). The BC showed to have a higher mean T_3_ concentration (122.9 ± 6.46; 95% CI: 105–140.9 ng⋅dL^− 1^) compared to the MBC (93.64 ± 10.75; 95% CI: 63.78–123.5 ng⋅dL^− 1^). The BC showed to have a higher mean Ghrelin concentration (91.63 ± 8.14; 95% CI: 69.01–114.2 pg⋅mL^− 1^) compared to the MBC (40.05 ± 5.71; 95% CI: 24.18–55.92 pg⋅mL^− 1^). The MBC showed to have a higher mean leptin concentration (25.55 ± .98; 95% CI: 22.81–28.28 ng⋅mL^− 1^) compared to BC (3.61 ± .17; 95% CI: 3.13–4.09 ng⋅mL^− 1^) and a higher normalized leptin to kg fat mass concentration (2.89 ± .24; 95% CI: 2.22–3.56 ng⋅mL^− 1^⋅kgFM^− 1^) compared to the BC (.37 ± .03; 95% CI: .28–.47 ng⋅mL^− 1^⋅kgFM^− 1^)

### Psychometrics Figs. 13 and 14

**Fig. 13:**
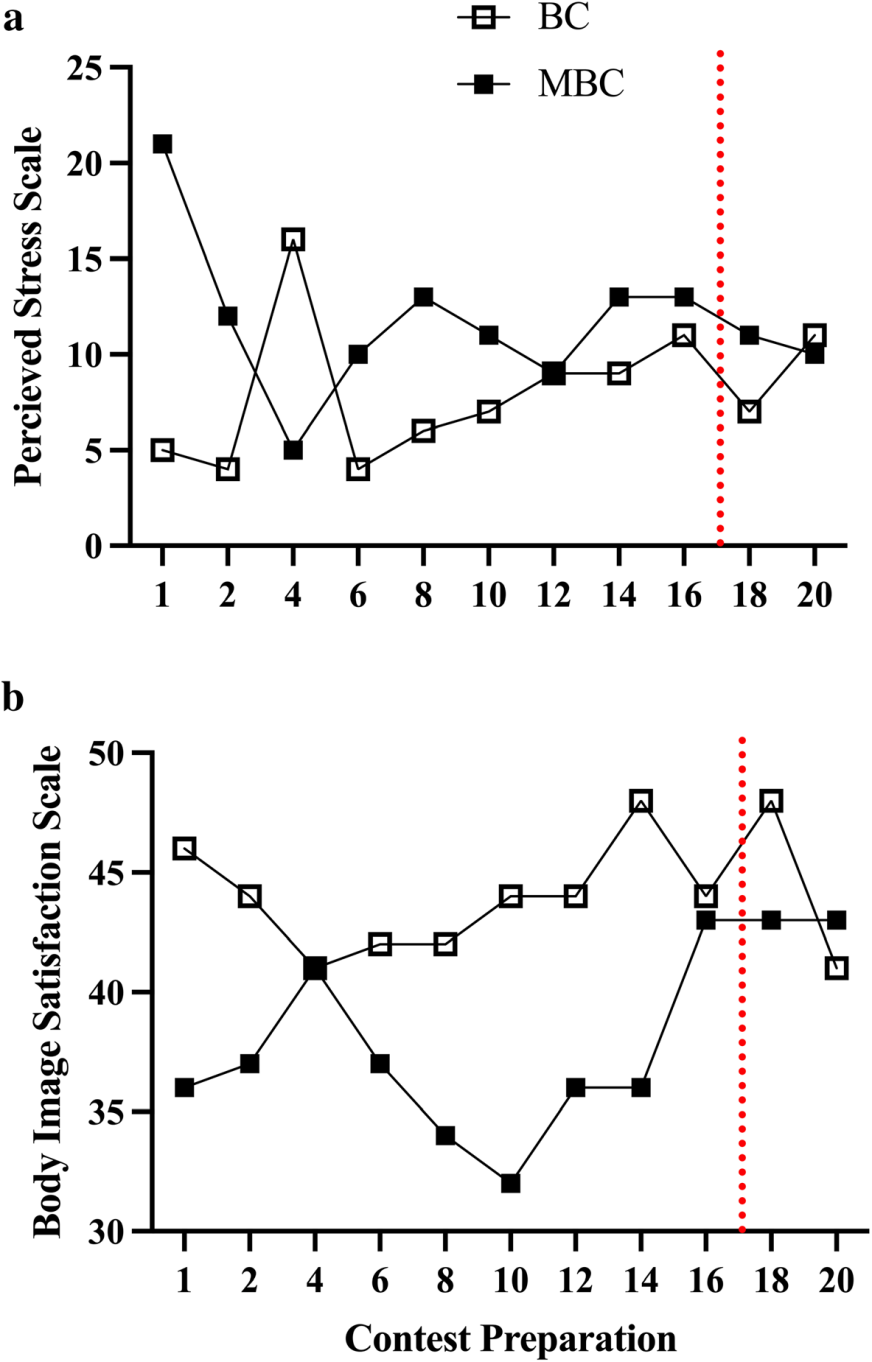
The time-course analysis of the impact of a 20-week contest preparation on the Perceived Stress Scale (PSS) (BISS) and the Body Image Satisfactory Scale with accompanying mean average (mean ± SEM; 95% CI:) comparison between BC and MBC on **a**) PSS, **b**) BISS. The MBC showed to have a higher mean PSS score (11.64 ± 1.17; 95% CI: 9.03–14.24) compared to the BC (8.09 ± 1.09; 95% CI: 5.66–10.52). The BC showed to have a higher mean BISS score (44.0 ± .75; 95% CI: 42.33–45.67) compared to the MBC (38.0 ± 1.16; 95% CI: 35.4–40.6). The red dashed line denotes competition

**Fig. 14:**
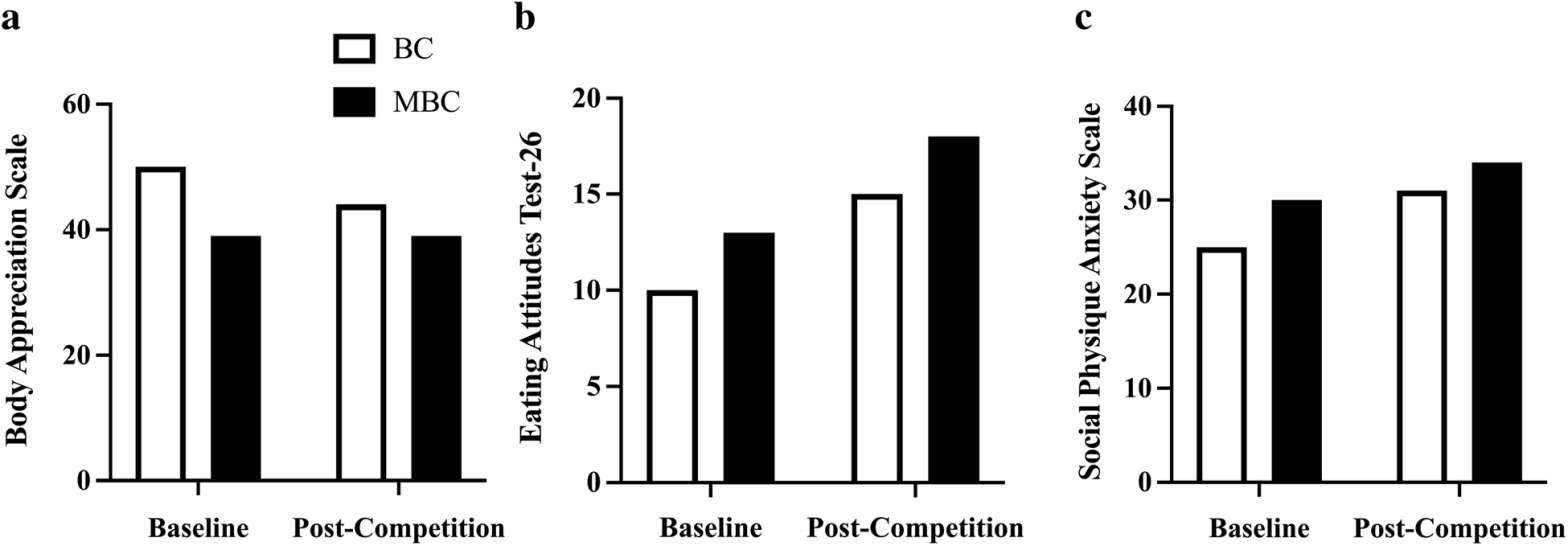
The psychometric analysis comparing baseline and pre-competition between BC and MBC on the **a**) Body appreciation scale (BAS), **b**) Eating attitude scale (EAT-26), and **c**) Social physique anxiety scale (SPAS) comparison between BC and MBC. The BC showed to have a higher BAS score at baseline and post-competition compared to the MBC’s baseline and post-competition values. Both the BC and the MBC scored higher on the EAT-26 post-competition compared to baseline. Both the BC and the MBC scored higher on the SPAS post-competition compared to baseline

## Discussion

Over the 16-week pre-contest preparation, as expected, both competitors lost ~ 4 kg of kg body weight, which was predominantly explained by a mean loss of ~ 3.7 kg of fat mass (FM) and a mean ~ 6% reduction in body fat (BF%). This suggests and assumes lean body mass (LBM) and skeletal muscle mass (SKMM) were comparatively well-conserved during contest preparation. The bikini competitor’s (BC) baseline value or starting point for both BF and BF% were both higher than the master’s bikini competitor (MPC) comparatively; however, the BC’s BF% change was much greater (~ 19%). This is concurrent with the BC’s greater reduction in ultrasound (US) assessed total subcutaneous adipose tissue (SAT) values with a − 34% reduction from baseline. Hulmi, et al. observed a ~ 7 kg fat mass loss from a higher fat mass baseline value (~ 14 kg) in their categorical division diverse female-physique population, which was more than double than seen in this bikini-physique categorical population. However, due to the diverse competitor division population assessed, it is difficult to determine the results for the bikini-physique competitors independently [12]. From our perspective, this may be explained in that these bikini-physique competitors maintained a higher BF% during their normal, non-contest preparation time periods (i.e.*,* “*offseason*”) compared to other notable female-physique competitor divisions with different judging criteria. These factors may determine the female-physique competitor’s goal of required fat mass for both offseason and optimal competition performance.

Prior to competing, and at their lowest fat mass assessment, both BC and MBC maintained their BF% greater than 12% (~ 14%), which is the recommended threshold for female athletes in weight-sensitive competition and sports to reduce risks of health defects [50]. Low BF% mixed with high energy expenditure and very low kilocalorie intake would lead to an LEA status. This has shown to negatively affect menstrual function, and bone mineral density, which may have clinical manifestations including eating disorders, functional hypothalamic amenorrhea, and osteoporosis known as “*The Female Athlete Triad*” [47]. In assessing both competitor’s LBM and interrelated SKMM measures, both were well preserved throughout their contest preparation (<Δ − 0.5 and − 1.68% respectively). It is well known that resistance training (RT) is a potent stimulator of muscle protein synthesis and muscle hypertrophy and concurrent with an energy-restricted state reduce LBM losses [51, 52]. Additionally, preservation of LBM in athletes is further increased with the combination of RT and higher protein intake [53] where it is was recommended an intake of 2.3–3.1 g·kg^− 1^ of fat-free mass (FFM) per day for energy-restricted, resistance training athletes [54]. Both the BC and MBC competitors reported a very high protein dietary intake at 2.96 ± .07 and 2.72 ± .05 g·kg^− 1^·d^− 1^ respectively during their contest preparation.

Both the BC and MBC showed fairly stable MF-BIA measured TBW, ICF, and ECF measures during the contest preparation. The BC showed a higher value of ICF fluid, which is most likely explained by a higher kg LBM and it is well-identified that ~ 60% of human TBW is stored intracellularly and represents 70–75% of LBM. We did not observe any notable change in TBW, ICF, and ECF measures 5 d post-competition in either the BC or the MBC. However, we did not acquire any dietary recall data post-competition. If the BC and MBC indulged in high kilocalorie, post-competition hyperphagia [26], it did not impact immediate TBW measures.

Our exploratory US measures, while not validated, did see some changes from baseline worth evaluating and interpreting due to their relative importance to the population assessed. In analyzing the regional deltoid (Delt_CSA_) muscle group in both competitors we saw an average increase in CSA (Δ + 4.63%) and reduction in gluteus maximus muscle thickness (GM_MT_) (Δ − 19.5%) muscle group. The BC showed to have a smaller GM_MT_ reduction (Δ-12.3%) compared to the MBC (Δ-26.7%). This dichotomy in muscular adaptations between the deltoid and the gluteus maximus is rather perplexing yet may be able to be partially explained by both exercise training and energy intake. Both competitors reported a similar mean % of lower body (LB) compared to upper body (UB) resistance training volume (BC: 52% UB; 48% LB and MBC: 49% UB; 51% LB). Overall, the BC reported having a much higher mean total RT volume (9194 ± 3499) compared to the MBC (5345 ± 1230 sets·reps). However, one session was not accurately reported and removed from the analysis. Unfortunately, we were unable to accurately relate the changes seen in Delt_CSA_ and GM_MT_ to the weekly volume of specific muscle group RT due to the inherent variation in both the BC’s and MBC’s self-reported training regimen (i.e.*,* not all training days and muscle groups were reported prior to the session). Interestingly, both the BC and MBC competitors self-reported using the Stairmill/Stairmaster as their primary mode of AT during their contest preparation. Both competitors reported an aerobic exercise frequency of ~ 6 d·week^− 1^ and over 4 d prior to their assessment an average of 198.9 ± 31.8 (95% CI:125.5–272.3) and 105.6 ± 23.9 (95% CI:48.99–162.3) min respectively where BC had a higher volume of AT. The selection of the Stairmill/Stairmaster as the primary choice mode of AT may have increased the overall work volume for the gluteus maximus muscle group. The gluteus maximus (GMax) is the largest muscle of the hip accounting for 16% of the total CSA in the region. This muscle group is often used to accelerate the body upward and forward from a position of hip flexion ranging from 45° to 60° (e.g.*,* pushing off into a sprint, arising from a deep squat, or climbing a very steep hill). It has been shown that the step-up exercise had the highest GMax myoelectrical activity (169.22 ± 101.47% MVIC) in comparison to other known hip exercises [55]. Although speculative, GM_MT_ reduction or atrophy may likely be more related to local glycogen and fluid loss than muscle protein loss due to restricted carbohydrate (CHO) intakes (BC: 3.64 ± .21 and MBC: 1.35 ± .15 g·kg^− 1^·d^− 1^), RT volume, and the selection of the Stairmaster/Stairmill as primary AT exercise of choice. To support fluid loss as a potential factor and a plausible explanation, in Fig. 7 we compared monthly Δ change with dual X-ray absorptiometry (iDXA) LBM (g), FM (kg) changes, and bioimpedance analysis (MF-BIA) configured total body water (TBW), extracellular fluid (ECF), and intracellular fluid (ICF) compartment changes (mL) over the 16-week pre-contest preparation period. Relationships between both LBM and TBW (r = .64; r [2] = .41; *p* = .04), and ECF (r = .72; r ^2^ = .52; *p* = .01) were found. No relationships were found between LBM and ICF changes, and no relationship was found between FM and TBW changes. Moreover, both BC and MBC showed an average ECF fluid loss over the 16 weeks. It has been shown that short-term hydration and muscle glycogen status may influence DXA-LBM measures [56–58]. Due to the inability of DXA to differentiate between ICF and ECF compartments, it is feasible that the GM_MT_ reduction may be potentially explained by local glycogen and fluid loss from concurrent LB exercise training volume, selected AT mode of exercise, and lower CHO intake.

In the exploration of utilizing the US to assess SAT measures and changes compared to iDXA, we found that time-course changes in the total of 7-site SAT measures correlated well with iDXA (r = .81; r^2^ = .66; *p* = .001) FM measures. Additionally, when estimating BF% using Jackson and Pollock [31] 7-site equation, we found US acquired SAT measures correlated well with iDXA configured BF% (r = .78; r^2^ = .60; *p* = .002). The US method has been previously utilized for SAT measures by Trexler, et al. that assessed physique athletes utilizing an automated program that estimated BF% [5]. However, we found no relationship between iDXA derived kg FM and US-SAT mm Δ changes. Utilizing the B-mode US as a modality to investigate SAT changes may have some practical use for individuals that do not have access to other expensive compositional measures to assess BF%.

No visual differences were observed between the BC and MBC total body water changes throughout the 20-week observational period. The MBC’s TBW saw a very slight reduction (− 1.3% Δ) compared to baseline values when comparing baseline to week 16. Additionally, we sought to assess both the BC’s and MBC’s TBW changes 5 d post-competition upon their return to the laboratory. Long periods of low energy status coupled with repetitive dietary choices and increased hunger may lead to immediate post-competition hyperphagia or binge eating [14] with an acute response similar to what may be seen with carbohydrate loading schemes used by endurance athletes after a glycogen depletion phase. Typically, glycogen is stored in a 1:3–4 ratio with water [59], which may lead to changes seen in weight gain and fluid perturbations post-competition. Both the BC and MBC saw no meaningful increases of either weight or TBW alterations 5-d post-competition.

In our observation of BMD, we did not expect to see any notable changes (~ ≤1% Δ change) in bone mineral density (BMD) or bone mineral content (BMC) over the 16-week contest preparation period due to the incremental effect of exercise on BMD to be very slow (6–12 mo) [60]. It should be noted, while no meaningful BMD changes were observed, both BC and MBC maintained > 100% of their age-related Z-score with lower observed E2 range levels. Further investigations should isolate the impact of resistance training-induced mechanotransductive stress compared to LEA-induced inhibition on reproductive hormone concentration and their integrated longitudinal effect on BMD in females.

During the 16-week contest preparation, both the BC and MBC maintained a mean categorical “*significant dehydrated*” state [37] (Fig. 8: BC: 1.021 ± .001 g·mL^− 1^ and MBC: 1.025 ± .001 g·mL^− 1^) where MBC averaged to be slightly more dehydrated than BC. However, from a practical aspect, it is unknown how meaningful this slight difference may be. It should be noted that each competitor was asked to visit the laboratory after an 8 h fast and prior to ingesting any food or drink. This USG assessment may not be indicative of their behaviors throughout the rest of the day and between visits. It is interesting after week 12, the BC’s hydration status moved below the 1.020 g·mL^− 1^, which is a status of euhydration [61]. It is unknown if the nearing competition influenced BC’s fluid intake and therefore hydration status. To our knowledge, we have not observed any previous literature examining the hydration status of competitors during their competition week. During this time, there may be manipulation of fluid consumption by restricting water intake [62] and/or pharmacologically induced fluid excretion through the use of diuretics [63] to reduce fluid content that may influence their ability to present muscular detail to the judging panel. Notably, due to the judging criteria for bikini-physique competitions, these water manipulating procedures may not be as aggressively used due to less focus on muscularity and conditioning. During competition week (D1-D5), comparatively, both the BC and MBC maintained euhydrated status during their competition (Fig. 8: D3, BC: 1.015 g·mL^− 1^; MBC 1.018 g·mL^− 1^ respectively). After the competition, the BC maintained a euhydrated status while the MBC’s values elevated closer to the average. This post-competition contrast in hydration status may be explained in that the BC chose not to compete in another competition and progress to an “off-season” status while the MBC elected to continue contest preparation to compete at another competition again a few weeks later.

During the competition, preparation saw a slight reduction in resting metabolic rate (RMR) at the 4-week time point for the BC. This may be explained by a self-reported reduction in kilocalorie intake and changes in body composition. Overall, during the contest preparation, RMR was fairly stable even with the observed body weight reduction where both BC and MBC showed mean positive Δ RMR change value. Our findings suggest that the majority of both BC’s and MBC’s kg weight loss was attributed to kg FM loss. We sought to analyze identify any relationship between change in Δ FM and Δ RMR. We found no correlation between contest preparation Δ FM change and Δ RMR change. However, it has been shown and readily accepted that the loss of both FM and LBM will impact RMR [64]. Additionally, in observing the active thyroid hormone triiodothyronine (T_3_) with its known relationship with metabolism, we observed no significant correlation with Δ T_3_ and Δ RMR change. In our results comparing baseline to week 16, we observed that both BC and MBC had a reduction in T_3_ hormone concentration of ~ 14 and 35% respectively. It is well established that thyroid hormone status regulates energy expenditure and therefore a factor in bodyweight changes [65]. Additionally, it has been shown that basal metabolic rate (BMR), which is ~ 10% lower than RMR is highly correlated with lean body mass. Being that LBM was mostly stable in both BC and MBC during contest preparation, this may suggest these competitor’s RMR was maintained by the factor of LBM more so than the observed T_3_ reduction. However, this is merely speculation being that none of these variables were assessed and isolated directly. Lastly, when comparing the BC and MBC competitors (Fig. 9) we found BC had a slightly higher mean RMR (+ 141 kcal·d^− 1^). However, when RMR values were normalized per kg LBM compared, a much smaller difference (~ 2.6 kcal·kgLBM^− 1^·d^− 1^) was found where BC was had a minimally higher RMR. This outcome was not entirely surprising in that there have been reports that a decline in RMR is associated with age. However, physically active older adults that maintain similar exercise training volume and energy intake maintain a similar RMR [66]. Moreover, it is known that LBM is highly correlated with RMR; however, visceral organ tissue is more metabolically active than is skeletal muscle tissue during resting conditions, which may explain some of the variances in RMR [67]. Therefore, in future studies assessing and comparing RMR in physique athletes, normalizing to LBM may reduce inherent variability. Lastly, it should be noted that the BC’s RMR increased post-competition at 20-week, after self-reporting following a “*reverse dieting*” protocol, a slight increase in FM (~ 1.1 kg) and reducing exercise training frequency and volume. The MBC continued a contest preparation regimen for another competition.

Our goal in observing endocrine responses in these bikini-physique competitors was to determine if age may have played a role in the responses to a restricted energy intake and increased energy expenditure. In our observation, estradiol (E2) and luteinizing hormone (LH) remained fairly stable for BC during contest preparation, with little variation in concentration from baseline to week 12. Both E2 and LH baseline values were near the lower end of both normal ranges. After the 12th week of the BC’s contest preparation, we observed a − 35% reduction of E2 at week 16, prior to competition. This concentration level fell below (~ 6 pg·mL^− 1^) what is considered the normal range relative to both follicular, mid-cycle, and luteal phases (10–300 pg·mL^− 1^). However, the BC’s mean E2 concentration over the 16-week contest preparation was 9.98 ± 1.73 pg·mL^− 1^ (95% CI: 5.43–14.35 pg·mL^− 1^). The mean average value falls within the range normally found in postmenopausal females (< 10 pg·mL^− 1^). Observing the BC’s LH time course, while relatively stable (3.66 ± .23 IU·L^− 1^; 95% CI: 3.00–4.31 IU·L^− 1^), LH concentration also reduced by ~ 13% at week 16 prior to the competition.

Comparatively, the MBC’s assessed E2 concentrations were more variable during the 16-week contest preparation. With the respect to the MBC’s age status of 44 y during this case series, which is close to the age of 45 y that has been shown in cross-sectional studies when endocrine changes and the onset of the perimenopause begin [68]. The MBC’s E2 concentrations reduced 24% from baseline values to week 16, prior to the competition. The mean average concentration during the 16-week pre-contest preparation was slightly less (9.31 ± 1.83 pg·mL^− 1^ (95% CI: 4.58–14.04 pg·mL^− 1^) than compared BC, yet also was observed below the normal concentration value found in postmenopausal women (< 10 pg·mL^− 1^).

The MBC’s LH time course concentration values seemed fairly stable until week 12 where there was a decline. There was an observed 14% reduction in LH when comparing baseline (95.9 IU·L^− 1^) and week 16 values (72.02 IU·L^− 1^). Interestingly, the MBC’s mean LH values over the 16-week pre-contest preparation were much higher than the BC (88.3 ± 13.4 vs 3.66 ± .23) IU·L^− 1^ respectively). This may be expected when taking into consideration that in early perimenopause, minor elevations in LH become evident [69].

In a seminal article by Loucks, et al. the “*energy availability hypothesis*” explained that LEA from low energy intake and high energy expenditure may inhibit gonadotropin-releasing hormone (GnRH) [70], which is derived from GnRH nerves located in the hypothalamic-pituitary-gonadal axis (HPTA) that is a pulse generator that controls the pulsatile secretion of the gonadotropic hormone LH, which is critical for reproduction [71]. Based on the BC’s dietary recall, the average energy intake over the 16-week pre-contest preparation was 43.2 ± 3.2 g·kgLBM^− 1^·d^− 1^. This value meets the recommended energy intake range requirement shown to maintain LH pulsatility [17]. However, due to the inability to accurately capture and quantify energy expenditure during this case series and then subtracting that value from energy intake, it is feasible to suggest that the actual energy availability may be lower than the estimated energy intake. In comparison, the MBC’s mean average, self-recalled dietary intake was 28.1 ± 1.1 g·kgLBM^− 1^·d^− 1^, which is below the recommended range to maintain LH pulsatility. Additionally, the self-reported value does not take into consideration energy expenditure from exercise training. This leads to an assumption that the energy availability may be lower for the MBC also. In contrast to Louck’s theory that suggests that LEA inhibits LH pulsatility [70], the higher LH concentrations observed during the MBC’s contest preparation may suggest that perimenopausal-induced LH increases may supersede LEA status inhibition of LH pulsatility. Furthermore, it should be noted that the majority of the work assessing LEA on female reproductive systems, has been investigated in younger, female athletes (< 29 y). We feel this is a fairly novel finding that requires much more investigation to determine how LEA may impact the reproductive system and metabolism in female athletes near perimenopause status.

Additional to assessing endocrine hormones related to reproduction and metabolism, we also investigated the impact of contest preparation on leptin and ghrelin. Leptin has been reported to influence various biological mechanisms such as initiating reproductive hormones, menstruation, regulatory centers in the brain to inhibit food intake and to regulate body weight and energy homeostasis [72]. Leptin is primarily synthesized and secreted from adipocytes in white adipose tissue and is normally found in higher blood concentrations in persons with higher BMI and BF%. Additionally, factors such as hyperglycemia and hyperinsulinemia also facilitate leptin secretion. However, in contrast, factors that are related to inhibiting leptin release are increasing age (≥40 y) [72]. In our investigation, we found that the BC’s leptin concentrations reduced 4% from baseline values at the 16-week time point. The BC’s mean average leptin concentration during pre-contest preparation fell slightly below the normal range (4.1–25.0 ng·mL^− 1^) in respect to BMI classification (3.6 ± .17 ng·mL^− 1^; 95% CI: 3.13–4.09 ng·mL^− 1^). The BC’s baseline leptin level was near the lower range normally found; however, this may be explained by a lower BF% compared to non-athlete females. The MBC’s mean average leptin levels were also within the normal range relative to BMI (22.8 ± .98 ng·mL^− 1^; 95% CI: 22.81–28.28 ng·mL^− 1^) yet were on the higher end of the normal scale compared to the BC. The MBC’s leptin levels time course was relatively stable during pre-contest preparation. Comparing baseline to the 16-week time period, there was a small ~ 1% increase found. This outcome was very intriguing and similar to the BC, the MBC also lost BF% from baseline to 16-weeks, yet this loss of fat mass did not seem to dictate leptin concentrations. This outcome is in contrast to Longstrom, et al. who observed leptin concentrations that were responsive to fat mass changes. However, it should be noted that the female-physique competitors observed in this study were ~ 29 y [8]. It may be plausible that there is a link between the elevated leptin and LH concentrations we observed in the MBC. It has been investigated in previous research that increased leptin appears to drive the reproductive system through both the HPTA and GnRH-stimulated LH secretion. Therefore, the increase seen in both leptin and LH in the MBC may be interrelated. Leptin directly stimulates ovarian steroidogenesis [73], yet, the E2 concentrations seemed to be less affected compared to LH found in the MBC. In our observation, it appears there may be some contrasting hormonal interrelationships between fat mass loss, leptin, LH, and E2 in our observations of the MBC compared to previous investigations that are typically seen in younger, female athletes.

Concurrent with assessing leptin responses in both BC and MBC, we sought to observe any differences in the hormone ghrelin, which is an orexigenic gut peptide. The fasted elevation of ghrelin levels and its decline after food ingestion led to its relevance as a ‘*hunger*’ hormone responsible for meal initiation, which is involved in the short-term regulation of food intake and long-term regulation of body weight through decreasing fat utilization [74]. Ghrelin has an impact on numerous physiological functions, although our focus was to observe any interrelationships with food intake and energy metabolism. In our observation of the BC’s ghrelin response was fairly stable throughout the 16-week pre-contest preparation. There was a notable − 36% drop in the BC’s ghrelin measure at week 4. This may be explained by the ~ 59% increase (kcal·kgLBM^− 1^·d^− 1^) that was self-reported from baseline to week 4 due to the ghrelin secretion being regulated by nutritional status. Interestingly, the BC’s mean ghrelin hormone concentration levels remained higher than the MBC throughout the 16-week pre-contest preparation (BC: 91.6 ± 8.1 vs MBC: 40.0 ± 5.7 pg·mL^− 1^; *p* = .0008) concurrent with a higher mean kcal·kgLBM^− 1^·d^− 1^ than the MBC. The MBC’s ghrelin level increased from ~ 70% from week 4 to week 8. However, there were no changes in self-reported dietary intake (kcal·kgLBM^− 1^·d^− 1^). Age is a factor that influences ghrelin secretion, which may assist in explaining the differences seen. However, the variations at certain time points may have other confounding factors influencing ghrelin concentration outside the parameters of our study. Our ghrelin findings and interpretations should be considered with much caution. It should be noted that the concentrations found in these bikini-physique competitors that were assessed using ELISA analysis were much lower than previous work assessing total ghrelin (both active acyl-ghrelin and inactive des-acyl-ghrelin) hormone with similar ELISA methodology (baseline ~ 500 pg·mL^− 1^) [75] and differing radioimmunoassay (~ 1625 pg·mL^− 1^) [76] protocol in healthy, normal, and similar BMI values as our bikini-physique competitors. Additionally, another factor that may explain such low values we observed is the half-life of acyl-ghrelin in human plasma without a stabilizer or deacylation inhibitor. Per our methodology, we used K_2_ EDTA vacutainer tubes for blood collection and stored plasma samples in a − 80 °C environment. However, previous investigations showed that fasting levels of plasma-derived-acyl-ghrelin collected in K_2_ EDTA vacutainers decreased approximately five-fold from prior storage measurements [77]. Future research that would like to investigate the gut-derived *hunger*’ hormone ghrelin in the blood may add this protocol to standard manufacturer ELISA methodology. To maintain sample integrity, the vacutainers may be treated with 4-(2-Aminoethyl) benzenesulfonyl fluoride hydrochloride (AEBSF) to reduce the degradation of ghrelin [77] to improve the accuracy of results.

In our psychometric findings, we observed that the MBC showed to have a higher mean average score of perceived stress (PSS) and a lower mean average score in the body image satisfactory (BISS) when compared to the BC. With notable environmental (e.g.*,* lifestyle, competition experience) and inherent psychological factors that could influence these measures, it is unknown if age has may have impacted these responses. The BAS-2 assessment pre- and post-contest preparation were stable for BC. In comparison, the MBC’s response declined from baseline to post-contest preparation. This difference found may be partially explained in that the MBC continued with contest preparation for another competition while the BC elected to progress into an “*off-season*” status. The eating attitude analysis (EAT-26) showed both BC and MBC increase post-competition when compared to baseline. This difference may be partially explained and influenced by post-competition hyperphagia. Lastly, the measures of social anxiety both increased from baseline to post-contest in the BC and MBC. The anxiety related to contest performance may play a role in this assessment.

## Conclusion

Our case series investigation of the 32 y and the master’s 44 y bikini-physique competitors during a 16-week pre-contest preparation observed that their adaptations were fairly similar in that no differences found in musculoskeletal or body water changes during pre-contest preparation. The hormonal differences seen may be explained due to a difference in age being the middle-aged bikini-physique competitor may be near perimenopausal status. Hydration status during pre-contest preparation was considered to be mainly in a dehydrated state for both competitors. Both seemed to become more hydrated as the competition date became closer and maintained a positive hydration status on the day of the competition. There were notable volume differences in training protocols; however, this inter-variability could be expected between competitors. Similarly, dietary regimens and energy intake did not fall within the recommended ranges, which seems to be a normal response in investigations observing female-physique competitors [3, 7, 11]. The exploratory protocols used to assess skeletal muscle changes in the bikini-physique competitors have not been validated for accuracy. However, the regional muscle groups assessed may hold a more relative reference value for bikini-physique competitors than the validated measures of *vastus lateralis* [32, 33, 78]. With the understanding of the limitations of this case series investigation, the hormonal leptin and LH outcomes that were observed in the master’s bikini-physique competitor that elevated regardless of reduced body fat and low energy status should be further investigated in similar female demographic populations. This outcome was in contrast to what has been previously seen in younger female athletes during LEA [17, 70, 79] and other case studies observing leptin changes female-physique competitors [8]. With the increased popularity of bikini-physique competitions and the similar compositional adaptations seen in the master’s bikini-physique competitor compared to the younger bikini-physique competitor, more studies should recruit and observe competitors in the master’s division as this may assist other females in the demographical area to engage in exercise and nutritional training protocols that may minimize the known physiological and metabolic changes associated with menopause transition.

## Data Availability

The datasets during and/or analyzed during the current study available from the corresponding author on reasonable request.

## Abbreviations

IFBB: International Federation of Bodybuilding
NPC: National Physique Committee
BMR: Basal metabolic rate
BMI: Body mass index
BMD: Bone mineral density
CHO: Carbohydrate
DXA/iDXA: Dual X-ray absorptiometry
LBM: Lean body mass
US: Ultrasound
MT: Muscle thickness
CSA: Cross-sectional area
T_3_: Triiodothyronine
RMR: Resting metabolic rate
LEA: Low energy availability
EA: Energy availability
EI: Energy intake
EEE: Exercise energy expenditure
MF-BIA: Multifrequency bioelectrical impedance analysis
T_4_: Thyroxine
BC: Bikini competitor
MBC: Master’s bikini competitor
RT: Resistance training
UB: Upper body resistance training
LB: Lower body resistance training
AT: Aerobic training
FM: Fat mass
SAT: Subcutaneous adipose tissue
Delt_CSA_: Deltoid cross-sectional area
GMax: Gluteus maximus
GM_MT_: Gluteus maximus muscle thickness
TBW: Total body water
ICF: Intracellular fluid
ECF: Extracellular fluid
USG: Urine specific gravity
PSS: Perceived Stress Scale
BISS: Body Image States Scale
BAS-2: Body Appreciation Scale
SPAS: Social Physique Anxiety Scale
EAT-26: Eating Attitudes Test 26
SKMM: Skeletal muscle mass
D_2_O: Isotopic deuterium dilution
ASLT: Appendicular soft lean tissue
SEE: Standard error of estimate
MHz: Megahertz
ACSM: American College of Sports Medicine
NIH: National Institute of Health
ICC: Intraclass correlation coefficient
CV%: Coefficient of variation (%)
MRI: Magnetic resonance imaging
PSIS: Posterior superior iliac spine
IT: Ischial tuberosity
DDH_2_O: Doubly distilled de-ionized water
RMR_Meas_: Measured RMR
RMR_Calc_: Estimated RMR
EDTA: Ethylenediaminetetraacetic acid
WHO: World Health Organization
ELISA: Enzyme-linked immunosorbent assay
LH: Luteinizing hormone
E2: Estradiol
GnRH: Gonadotropin-releasing hormone
HPTA: Hypothalamic–pituitary-gonadal axis
AEBSF: 4-(2-Aminoethyl) benzenesulfonyl fluoride hydrochloride
